# Increased 15-PGDH expression leads to dysregulated resolution responses in stromal cells from patients with chronic tendinopathy

**DOI:** 10.1038/s41598-017-11188-y

**Published:** 2017-09-08

**Authors:** Stephanie G. Dakin, Lucy Ly, Romain A. Colas, Udo Oppermann, Kim Wheway, Bridget Watkins, Jesmond Dalli, Andrew J. Carr

**Affiliations:** 1 Nuffield Department of Orthopaedics, Rheumatology and Musculoskeletal Sciences, Botnar Research Centre, University of Oxford, Nuffield Orthopaedic Centre, Headington, OX3 7LD UK; 2 Lipid Mediator Unit, William Harvey Research Institute, Barts and The London School of Medicine and Dentistry, Queen Mary University of London, Charterhouse Square, London, EC1M 6BQ UK; 3 Structural Genomics Consortium, University of Oxford, Old Road Campus, Headington, OX3 7DQ UK

## Abstract

The mechanisms underpinning the failure of inflammation to resolve in diseased musculoskeletal soft tissues are unknown. Herein, we studied bioactive lipid mediator (LM) profiles of tendon-derived stromal cells isolated from healthy donors and patients with chronic tendinopathy. Interleukin(IL)-1β treatment markedly induced prostaglandin biosynthesis in diseased compared to healthy tendon cells, and up regulated the formation of several pro-resolving mediators including 15-epi-LXA_4_ and MaR1. Incubation of IL-1β stimulated healthy tendon cells with 15-epi-LXA_4_ or MaR1 down-regulated PGE_2_ and PGD_2_ production. When these mediators were incubated with diseased cells, we only found a modest down regulation in prostanoid concentrations, whereas it led to significant decreases in IL-6 and Podoplanin expression. In diseased tendon cells, we also found increased 15-Prostaglandin Dehydrogenase (15-PGDH) expression as well as increased concentrations of both 15-epi-LXA_4_ and MaR1 further metabolites, 15-oxo-LXA_4_ and 14-oxo-MaR1. Inhibition of 15-PGDH using either indomethacin or SW033291 significantly reduced the further conversion of 15-epi-LXA_4_ and MaR1 and regulated expression of IL-6, PDPN and STAT-1. Taken together these results suggest that chronic inflammation in musculoskeletal soft tissues may result from dysregulated LM-SPM production, and that inhibition of 15-PGDH activity together with promoting resolution using SPM represents a novel therapeutic strategy to resolve chronic tendon inflammation.

## Introduction

Tendinopathy and other soft tissue diseases are a common global disease burden causing pain and prolonged disability, and an increasing component of health expenditure in ageing societies^1, 2^. Multiple therapies have been advocated to treat tendinopathy including physiotherapy, non-steroidal anti-inflammatory drugs (NSAIDs), and local injections of glucocorticoids. As disease progresses, tendons may tear or rupture^3^ causing considerable pain and incapacity, necessitating surgical repair, which is frequently associated with high post-operative failure rates^4^. There are currently no effective treatments for patients with non-resolving tendinopathy that address the underlying biology of disease.

The etiology of tendinopathy is multifactorial, encompassing effects of repetitive overuse, aging and genetic factors^5, 6^. Growing evidence supports the contribution of inflammation to the onset and progression of disease^7–9^, however the mechanisms underpinning development of chronic tendon inflammation are unknown. Whilst immune cells including macrophages and T cells are recognised contributors to the inflammatory process^7, 9, 10^, the relative contributions of tendon cells (resident stromal fibroblasts) to sustaining inflammation are understudied. We previously investigated inflammation activation pathways in cultured stromal cells derived from human tendons, demonstrating that stromal cells derived from patients with tendinopathy may be ‘primed’ for inflammation^9^. Tissues and cells derived from patients with tendinopathy show increased expression of markers of stromal fibroblast activation including Podoplanin (PDPN), VCAM-1 (CD106) and Endosialin (CD248) compared to healthy tendon tissues and cells^11^. Stromal fibroblast activation is a feature of Rheumatoid Arthritis (RA) in which resident stromal cells fail to switch off their inflammatory programme. These phenotypic alterations in RA synovial fibroblasts play an important role in the switch from resolving inflammation to persistent disease^12, 13^. Collectively, these studies support the concept that resident stromal fibroblasts are implicated in the persistence of chronic inflammation, although the mechanisms underpinning the failure of inflammation to resolve are not understood.

Inflammation resolution is an active and highly coordinated process whereby a repertoire of pro-resolving lipid mediators and proteins promote the timely resolution of inflammation after injury and/or infection^14–16^. Perturbed resolution is thought to contribute to the development of many systemic chronic inflammatory diseases^17, 18^. Proresolving lipid mediators are well studied in experimental mouse models of systemic inflammation^19, 20^ as well as in humans^21, 22^. Evidence for their protective roles in chronic inflammatory diseases is growing, including periodontal disease^23^, inflammatory arthritis^24^ and pulmonary fibrosis^25^. Receptors implicated in mediating the effects of proresolving lipid mediators including the lipoxin A_4_ receptor ALX/FPR2 and the Resolvin E1 receptor ERV1/ChemR23 have been identified in diseased human tendons^9^, suggesting a role for these mediators in disease etiopathology. Of note, to date the presence of these pro-resolving mediators and their regulation in diseased human tendon cells remains of interest.

The present study focused on identification of mechanisms underpinning the development of chronic inflammation in diseased human tendon tissues, which are currently poorly understood. We utilised an omics approach to perform a comprehensive analysis of pro-inflammatory and pro-resolving lipids in cultures of stromal fibroblasts derived from healthy and diseased human tendons. Using lipid mediator profiling, we identified differences in bioactive lipid mediator profiles between healthy and diseased tendon-derived stromal cells after treatment with IL-1β. We also investigated the biological actions of proresolving lipid mediators 15-epi-LXA_4_ and MaR1 on counter-regulating dysregulated resolution processes in diseased tendon cells. The findings from this study provide improved understanding of the biological roles of SPM in diseased musculoskeletal soft tissues. We identify a mechanism underpinning dysregulated resolution responses in stromal cells from patients with tendinopathy, and propose a novel therapeutic strategy to promote resolution of chronic tendon inflammation.

## Results

### Diseased tendon-derived stromal cells display dysregulated resolution responses

**Figure 1:**
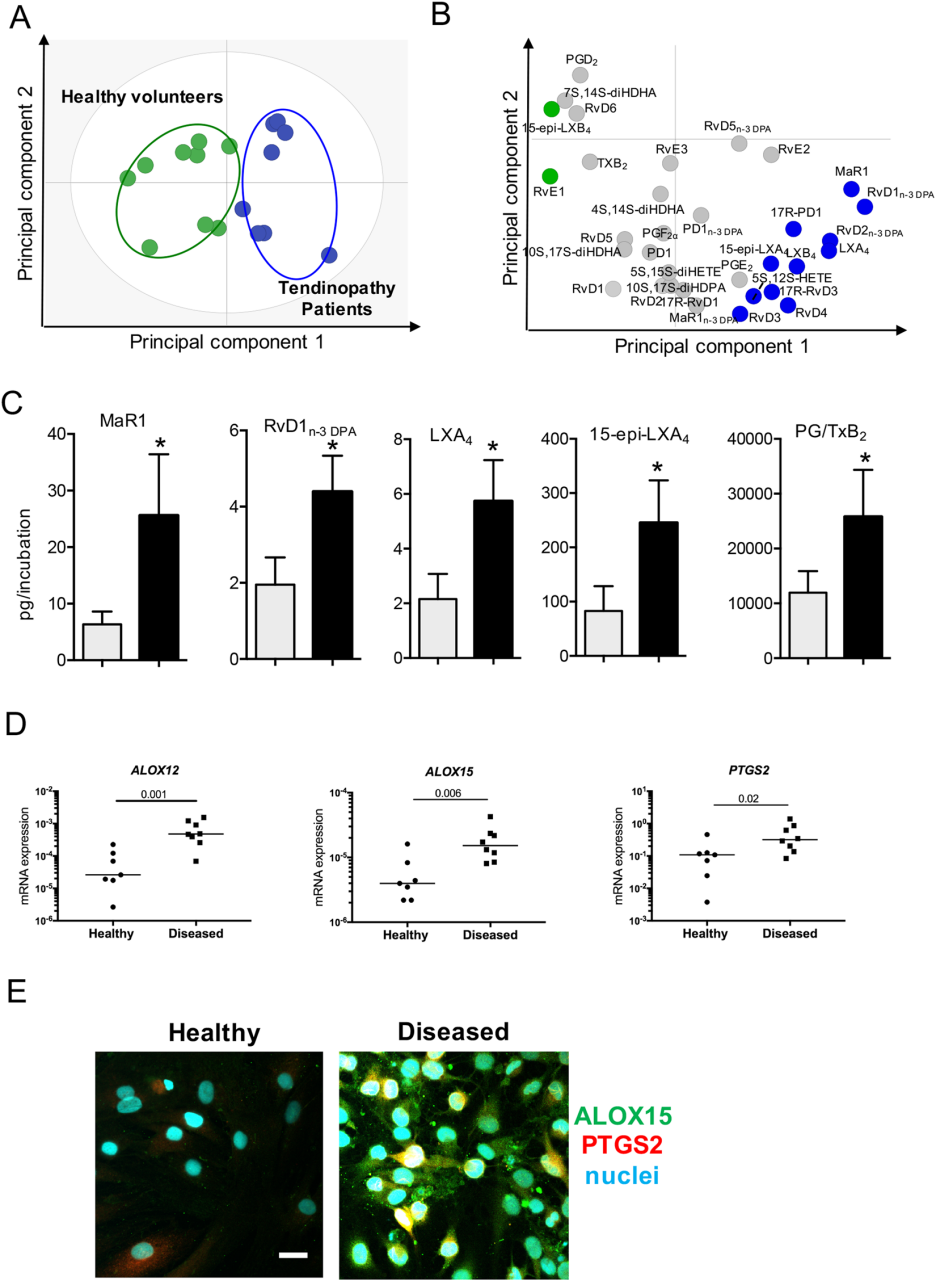
Distinct SPM profiles in IL-1β stimulated healthy and diseased tendon stromal cells. Tendon stromal cells (60,000 cells per well) were derived from healthy hamstring (n = 8 donors) or diseased supraspinatus tendons (n = 8 donors). Cells were cultured in DMEM F12 phenol red free medium containing 1% heat inactivated human serum to 80% confluence and incubated with IL-1β for 24 h. Media and cells were harvested and placed in ice-cold methanol containing deuterium labeled internal standards. LM were then extracted and profiled. (**A**) 2-dimensional score plot and (**B**) corresponding 2-dimensional loading plot of plasma LM-SPM from human tendon derived-stromal cell incubations isolated from healthy volunteers or patients with tendinopathy after stimulation with IL-1β (10ngml^—1^) for 24 h. Grey ellipse in the score plot denotes 95% confidence regions. (**C**) Concentrations for mediators found to be differentially regulated between healthy (grey bars) and diseased (black bars) tendon stromal cell incubations. Results are shown as means and SEM and representative of n = 8 donors per group. (**D**) mRNA expression of lipid mediator biosynthetic enzymes determines using real time qPCR. Gene expression is normalized to β-actin, bars show median values. (**E**) Representative immunofluorescence images showing staining for ALOX15 (green), PTGS2 (red) and nuclei (cyan) in IL-1β-stimulated healthy and diseased tendon cells. Scale bar, 20μm. Results are representative of n = 3 donors.

Lipid mediator (LM) profiling of healthy hamstring and diseased supraspinatus tendon-derived stromal cell cultures identified specialized pro-resolving lipid mediators (SPM) including D-series Resolvins (RvD1, RvD2, RvD3, RvD4, RvD5, RvD6, 17R-RvD1 and 17R-RvD3), Protectins (PD1, 17R-PD1), Maresins (MaR1), E-series Resolvins (RvE1, RvE2, RvE3), arachidonic acid-derived Lipoxins (LXA_4_, LXB_4_, 15-epi-LXA_4_ and 15-epi-LXB4) and n-3 DPA-derived Resolvins RvD1_n-3 DPA_, RvD2_n-3 DPA_ and RvD5_n-3 DPA_), Protectins (10 S,17S-diHDPA) and Maresins (MaR1_n-3 DPA_). These mediators were identified in accordance with published criteria that include matching retention times and at least 6 ions in the tandem mass spectrum^26^ (Fig. 1A,B, Supplementary Figure 1). Multivariate analysis uncovered differences in bioactive LM profiles between healthy and diseased tendon cells following incubation with IL-1β (10ngml^−1^) for 24 hours as demonstrated by the distinct clustering of the LM profiles (Fig. 1A and B). Assessment of individual LM concentrations demonstrated significant increases in several SPM including Maresin (MaR) 1, n-3 DPA derived D-series resolvin (RvD1_n-3 DPA_), LXA_4_ and 15-epi-LXA_4_. In these incubations we also found significant increases in several inflammation initiating eicosanoids including PGE_2_ (Fig. 1C and Table 1). This increase in both SPM and eicosanoids in tendon derived stromal cells from patients with tendinopathy was coupled with a significant increase in the expression of several of their biosynthetic enzymes including *ALOX12, ALOX15* and *PTGS2* (Fig. 1D and E). These findings suggest that although SPM are up regulated in stromal cells from patients with tendinopathy, their concentrations are not sufficient to counter regulate the ongoing inflammatory processes, reminiscent of a dysregulated resolution response characteristic of chronic inflammatory conditions^27–29^.

**Table 1:** Altered LM profiles of diseased tendon stromal cells when compared to those from healthy tendon stromal cells.

DHA bioactive metabolome	Q1	Q3	Tendon stromal cells Lipid mediators levels (pg/incubation)	
			Healthy + IL-1β	Disease + IL-1β
RvD1	375	141	5.3 ± 1.8	4.6 ± 2.0
RvD2	375	141	8.7 ± 4.3	11.6 ± 5.3
RvD3	375	147	4.1 ± 2.5	6.6 ± 2.7
RvD4	375	101	4.2 ± 2.1	7.9 ± 3.3
RvD5	359	199	53.9 ± 11.1	49.3 ± 7.1
RvD6	359	101	24.1 ± 7.7	13.9 ± 2.7
17R-RvD1	375	141	1.9 ± 0.6	2.1 ± 0.6
17R-RvD3	375	147	5.7 ± 1.7	7.6 ± 2.1
PD1	359	153	27.6 ± 5.8	26.6 ± 9.5
17R-PD1	359	153	1.0 ± 0.2	1.7 ± 1.0
10 S,17S-diHDHA	359	153	139.7 ± 10.3	117.1 ± 41.3
MaR1	359	221	6.3 ± 2.4	28.7 ± 11.8*
7 S,14S-diHDHA	359	221	40.5 ± 22.3	7.9 ± 2.9
4 S,14S-diHDHA	359	101	15.8 ± 6.8	19.8 ± 6.9
**n-3 DPA bioactive metabolome**				
RvD1_n-3 DPA_	377	143	2.0 ± 0.8	4.8 ± 1.0*
RvD2_n-3 DPA_	377	261	0.7 ± 0.6	2.3 ± 1.3
RvD5_n-3 DPA_	361	263	19.9 ± 4.3	23.0 ± 5.9
PD1_n-3 DPA_	361	183	58.0 ± 26.4	98.6 ± 54.0
10 S,17S-diHDPA	361	183	414.3 ± 207.1	499.3 ± 263.5
MaR1_n-3 DPA_	361	249	13.8 ± 5.6	13.3 ± 4.5
**EPA bioactive metabolome**				
RvE1	349	195	0.3 ± 0.2	0.1 ± 0.1
RvE2	333	199	39.8 ± 10.1	54.3 ± 6.0
RvE3	333	201	2.0 ± 1.1	2.0 ± 0.9
**AA bioactive metabolome**				
LXA_4_	351	217	2.2 ± 1.0	6.0 ± 1.7*
LXB_4_	351	221	40.9 ± 18.5	96.2 ± 52.9
5 S,15S-diHETE	335	235	1404.0 ± 358.7	1727.2 ± 437.5
15epi-LXA_4_	351	217	82.8 ± 48.4	245.2 ± 78.3*
15epi-LXB_4_	351	221	57.7 ± 16.8	43.6 ± 12.4
15-oxo-LXA_4_	349	233	0.5 ± 0.2	3.0 ± 1.3*
LTB_4_	335	195	57.0 ± 49.4	0.0 ± 0.0
5 S,12S-diHETE	335	195	138.8 ± 63.9	264.8 ± 88.0
PGD_2_	351	189	2065.8 ± 906.4	968.0 ± 294.1
PGE_2_	351	189	9483.0 ± 4429.1	21371.2 ± 8192.9
PGF_2α_	353	193	367.0 ± 79.8	380.1 ± 102.5
TxB_2_	369	169	20.8 ± 5.8	12.1 ± 2.2

Tendon stromal cells (60,000 cells per well) were derived from healthy hamstring (n = 8 donors) or diseased supraspinatus tendons (n = 8 donors). Cells were incubated for 24 h with IL-1β, incubations were quenched using ice-cold methanol containing deuterium labelled internal standards and lipid mediators (LM) were identified and quantified using LM profiling (see methods for details). Q1, M-H (parent ion) and Q3, diagnostic ion in the MS-MS (daughter ion). Results are expressed as pg/incubation. Mean ± SEM of n = 8 per incubation. *P < 0.05 vs Healthy. The detection limit was ~ 0.1 pg. -, Below levels found in media alone.

### 15-epi-LXA_4_ and MaR1 up regulate SPM production and reduce inflammatory responses in both diseased and healthy tendon-derived stromal cells

**Figure 2:**
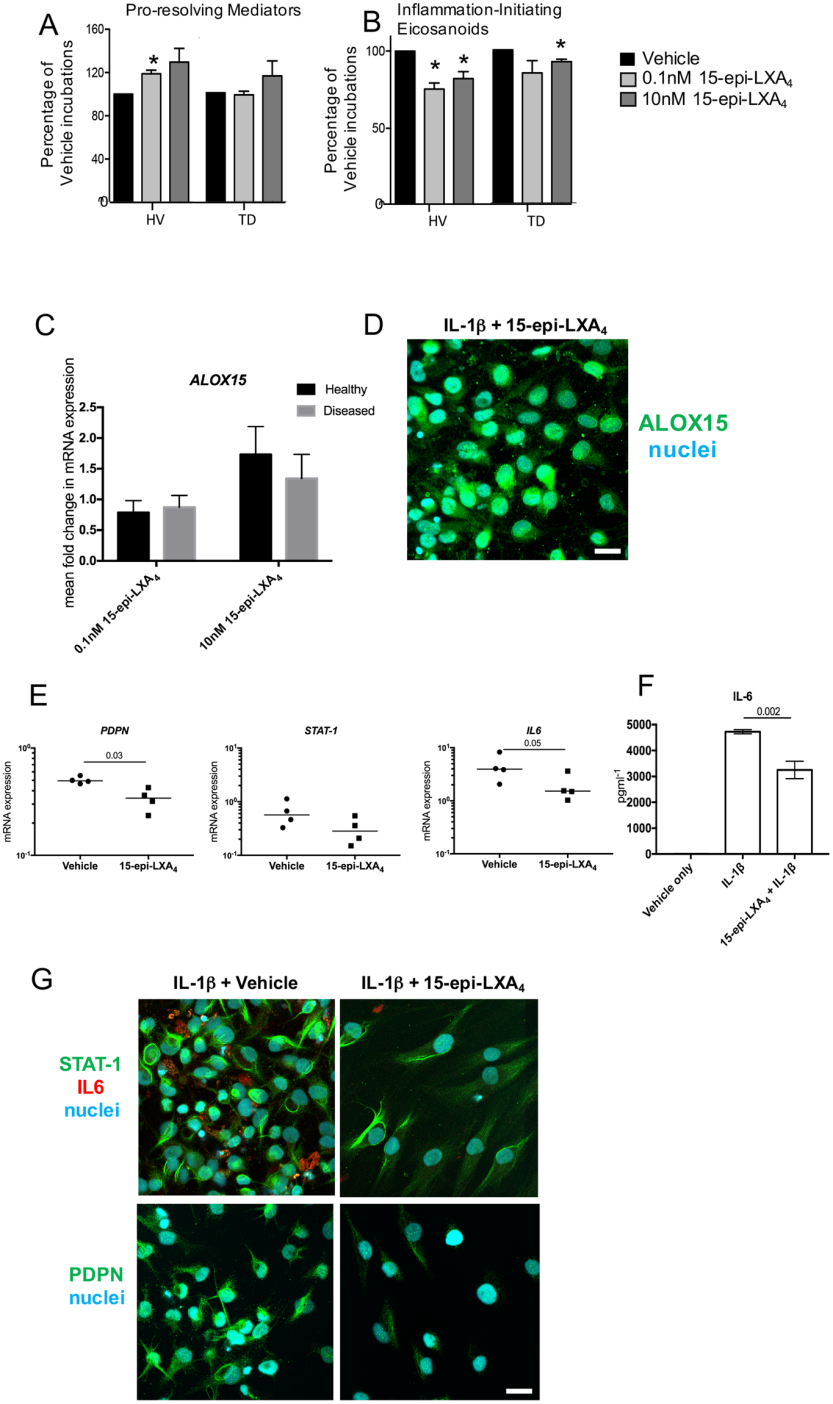
15-epi-LXA_4_ up regulates SPM and decreases pro-inflammatory mediators in healthy and diseased tendon stromal cells. Tendon stromal cells were derived from healthy hamstring (H, n = 7 donors) or diseased supraspinatus tendons (TD, n = 6 donors). Cells were incubated with 15-epi-LXA_4_ (0.1 or 10 nM) or Vehicle for 24 h at 37 °C then with IL-1β (10ngml^—1^) for 24 h. (**A**,**B**) LM were identified and quantified using LM profiling (see methods for details). Cumulative levels (**A**) DHA-derived (RvD, PD, MaR) n-3 DPA-derived (RvD_n-3 DPA_, PD_n-3 DPA_, MaR1_n-3 DPA_), EPA-derived (RvE) and AA-derived (LX) pro-resolving mediator levels. (**B**) Pro-inflammatory eicosanoids (PG, Tx) in tendon stromal cells from healthy volunteers (HV) and patients with tendinopathy (TD). Statistically significant differences were calculated using pairwise Mann-Whitney U tests. *p < 0.05 vs Vehicle incubations. (**C**) Incubation of IL-1β stimulated diseased tendon cells in 0.1 or 10 nM 15-epi-LXA_4_ induced expression of *ALOX15* mRNA relative to vehicle controls. (**D**) Immunocytochemistry for ALOX15 in IL-1β stimulated diseased tendon stromal cells incubated in 10 nM 15-epi-LXA_4_. (**E**) mRNA expression of *PDPN*, *STAT-1* and *IL-6* determined using quantitative RTPCR. Gene expression is normalized to β-actin, bars show median values. (**F**) ELISA assay of IL-6 protein secretion from IL-1β stimulated diseased tendon cells incubated in the presence and absence of 10 nM 15-epi-LXA_4_. Data are shown as means and SEM, n = 4 separate donors. (**G**) Representative immunofluorescence images showing staining for STAT-1 (green), IL-6 (red), PDPN (green), and nuclei (cyan) in IL-1β stimulated diseased tendon stromal cells incubated in 10 nM 15-epi-LXA_4_. All images are representative of n = 3 donors. Scale bar, 20 μm.

Having found altered LM-SPM profiles following addition of IL-1β, we next tested whether addition of 15-epi-LXA_4_ and MaR1, two of the mediators up regulated in diseased tendon stromal cell incubations (Fig. 1), modulated responses in stromal cells isolated from healthy hamstring and diseased supraspinatus tendons. Incubation of tendon-derived stromal cells with either 0.1 nM or 10 nM 15-epi-LXA_4_ dose-dependently up regulated SPM production, including the DHA derived RvD, PD and MaR as well as the EPA derived E-series resolvins in healthy volunteer cell incubations (Fig. 2A, Supplemental Table 1). In addition, in these cell incubations we also found dose dependent decreases in the concentrations of inflammation initiating prostaglandins (PG), primarily PGE_2_, a mediator that carries both pro-inflammatory and nociceptive actions^30^ (Fig. 2B, Supplemental Fig. 2 and Supplemental Table 1). Of note, when 15-epi-LXA_4_ was incubated with cells derived from patients with tendinopathy, these cells displayed blunted actions in up regulating SPM production (Fig. 2, Supplemental Fig. 2, Supplemental Table 2). We also found a dose dependent decrease in PG levels, however these decreases were less pronounced than those observed with cells from healthy volunteers. Moreover, 15-epi-LXA_4_ induced expression of *ALOX15* mRNA relative to IL-1β-stimulated vehicle controls (Fig. 2C) and induced ALOX15 protein in diseased tendon stromal cells (n = 3 donors) (Fig. 2D).

We next assessed whether 15-epi-LXA_4_ also regulated other markers of tendon inflammation in patient-derived stromal cells. Incubation of IL-1β-stimulated diseased tendon cells with 10 nM 15-epi-LXA_4_ reduced PDPN mRNA (p = 0.03) and protein, moderated STAT-1 mRNA and protein and reduced IL-6 mRNA and protein levels in tissue culture media (p = 0.002) (Fig. 2E–G).

**Figure 3:**
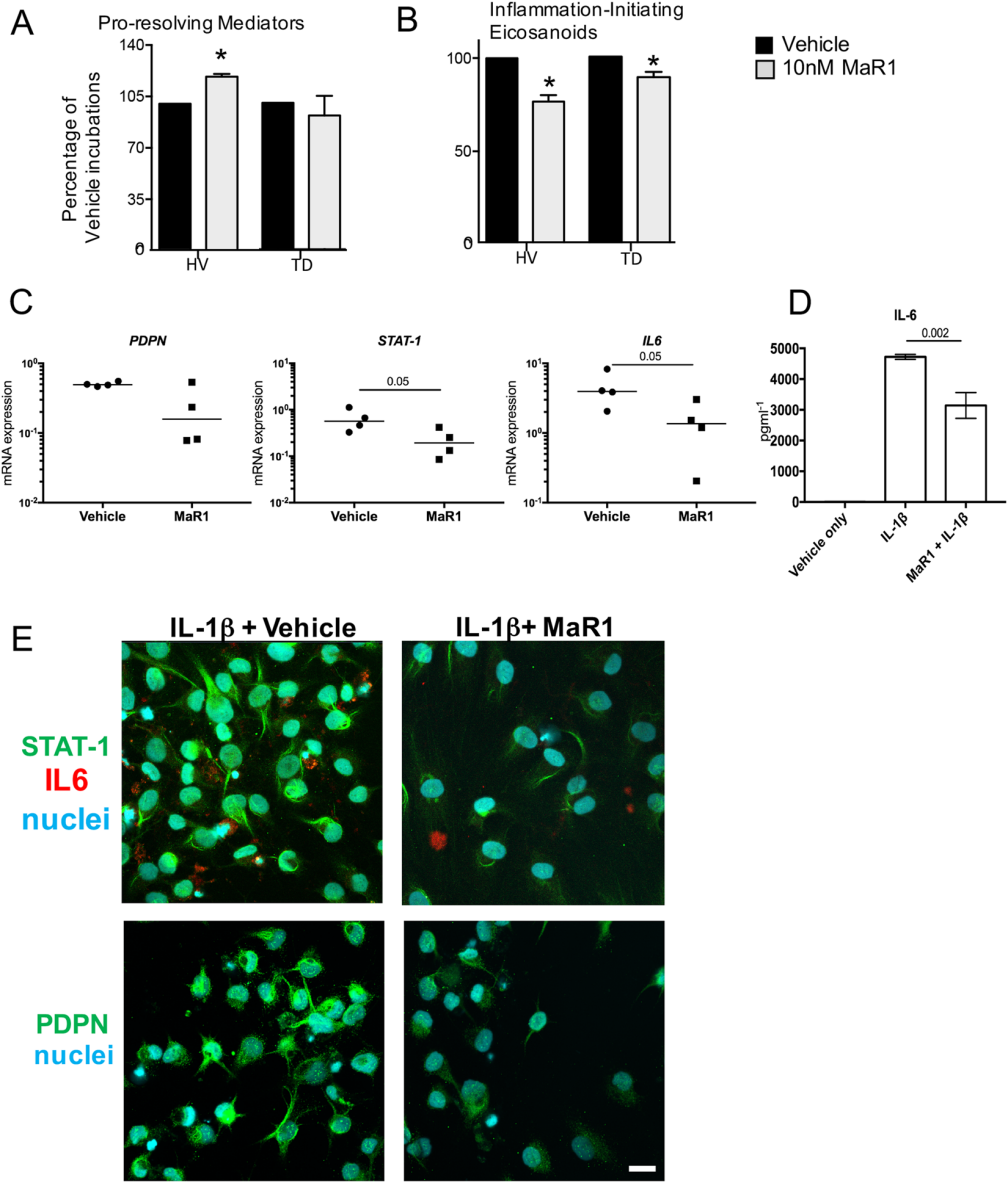
MaR1 counter regulates the inflammatory actions of IL-1β in healthy and diseased tendon stromal cells. Tendon stromal cells were derived from healthy hamstring (H, n = 7 donors) or diseased supraspinatus tendons (TD, n = 6 donors). Cells were incubated with MaR1 (10 nM) or Vehicle for 24 h at 37 °C then with IL-1β (10ngml^—1^) for 24 h. **(A**,**B)** LM were identified and quantified using LM profiling (see methods for details). Cumulative levels **(A)** DHA Derived (RvD, PD, MaR) n-3 DPA-derived (RvDn-3 DPA, PDn-3 DPA, MaR1n-3 DPA), EPA-derived (RvE) and AA-derived (LX) proresolving mediator levels. (**B**) Pro-inflammatory eicosanoids (PG, Tx) in tendon stromal cells from healthy volunteers (HV) and patients with tendinopathy (TD). Statistically significant differences were calculated using pairwise Mann-Whitney U tests. *p < 0.05 vs Vehicle incubations. (**C**) mRNA expression of *PDPN*, *STAT-1* and *IL-6* determined using quantitative RTPCR. Gene expression is normalized to β-actin, bars show median values. (**D**) ELISA assay of IL-6 protein secretion from IL-1β stimulated diseased tendon cells incubated in the presence and absence of 10 nM MaR1. Data are shown as means and SEM, n = 4 separate donors. (**E**) Representative immunofluorescence images showing staining for STAT-1 (green), IL-6 (red), PDPN (green), and nuclei (cyan) in IL-1β stimulated diseased tendon stromal cells incubated in 10 nM MaR1. All images are representative of n = 3 donors. Scale bar, 20 μm.

Incubation of tendon-derived stromal cells with 10 nM MaR1 also significantly up regulated SPM concentrations in incubations with healthy cells. Here we found increases in RvD and LX as well as statistically significant decreases in inflammation initiating eicosanoids including PGE_2_ (Fig. 3A and B, Supplemental Fig. 3 and Supplemental Table 1). Incubation of diseased tendon cells with MaR1 also lead to a decrease in the levels of PG and Tx, although as observed for 15-epi-LXA_4_ the reduction in these pro-inflammatory eicosanoids was less than that observed with cells from healthy volunteers (Fig. 3, Supplemental Fig. 3 and Supplemental Table 2). In addition to regulating eicosanoid production, incubation of MaR1 with diseased tendon cells also lead to down-regulation of mRNA and protein of PDPN, STAT-1 and IL-6 (Fig. 3C–E). Together these findings demonstrate that MaR1 and 15-epi-LXA_4_ counter regulate IL-1β initiated inflammation in tendon-derived stromal cells. They also point to a dysregulated resolution response in cells derived from patients with tendinopathy, given the lower potency of these mediators at regulating the production of both pro-resolving and pro-inflammatory mediators.

### Cells from tendinopathy patients display enhanced ability for further conversion of SPM to metabolites that carry reduced biological actions

**Figure 4:**
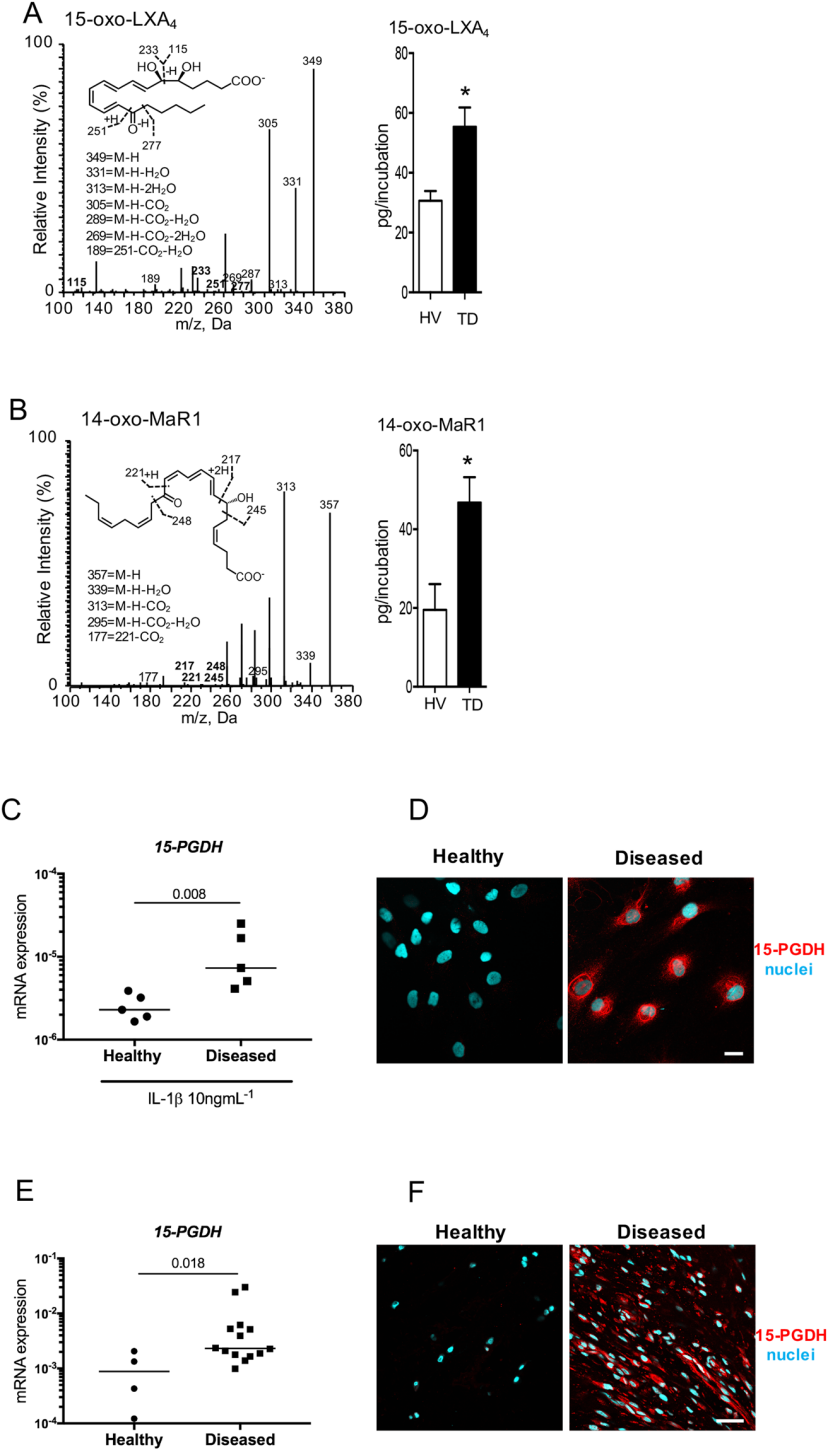
Enhanced further metabolism of 15-epi-LXA_4_ and MaR1 in diseased tendon stromal cells. Cells were incubated with 10 nM of 15-epi-LXA_4_, MaR1 or vehicle for 24 h at 37 °C then with IL-1β for 24 h at 37 °C. At the end of the incubations 2 volumes of ice-cold methanol were added and products were identified and quantified using LM profiling (see methods for details). (**A**) MS-MS spectrum employed for the identification of 15-oxo-LXA_4_ (left panel); and 15-oxo-LXA_4_ concentration in stromal cell incubations from healthy volunteers (HV) and patients with tendinopathy (TD; right panel) **(B**) MS-MS Spectrum employed for the identification of 14-oxo-MaR1 (left panel); and 14-oxo-MaR1 concentration in stromal cell incubations from HV and TD (right panel). Statistically significant differences were calculated using pairwise Mann-Whitney U tests. Results are presented as means ± SEM. *P ≤ 0.05, n = 7 donors for HV and 6 donors for TD. (**C**) 1*5-PGDH* mRNA expression in stromal cells derived from healthy hamstring (n = 5) and diseased supraspinatus tendons (n = 5) after stimulation with IL-1β (10ngml^—1^) for 24 h. Statistically significant differences were calculated using pairwise Mann-Whitney U tests. Gene expression is normalized to β-actin, bars show median values. (**D**) Representative immunofluorescence images of tendon stromal cells isolated from healthy hamstring donors (n = 3) and patients (n = 3) with supraspinatus tendinopathy showing staining for 15-PGDH (red) and nuclei (cyan) after stimulation with IL-1β (10ngml^—1^) for 24 h. (**E**) 1*5-PGDH* mRNA expression in healthy subscapularis (n = 4 donors) and diseased supraspinatus (n = 14 donors) shoulder tendon tissues. Gene expression is normalized to β-actin, bars show median values. **(F**) Representative immunofluorescence images of sections of diseased and healthy shoulder tendons stained for 15-PGDH (red). Cyan represents POPO-1 nuclear counterstain. Scale bars, 20μm.

Having determined that the biological actions of 15-epi-LXA_4_ and MaR1 were blunted in tendon cells derived from donors with tendinopathy, we next investigated the mechanism underpinning this observation. Given that SPM further metabolism may lead to their inactivation^31^, we investigated SPM further metabolism of 15-epi-LXA_4_ and MaR1 in IL-1β-stimulated tendon cells derived from healthy (n = 7) and diseased (n = 6) donors incubated in either 10 nM 15-epi LXA_4_ or 10 nM MaR1. In incubations of diseased or healthy tendon cells with IL-1β and 15-epi-LXA_4_ (10 nM), we identified the inactive LX further metabolite 15-oxo-LXA_4_ (Fig. 4A)_._ Concentrations of this inactive LX further metabolite were elevated in diseased cells compared to healthy cells (p = 0.01) (Fig. 4A, Supplemental Tables 1 and 2). Similarly, in incubations with cells from tendinopathy patients, IL-1β and MaR1 (10 nM) we found elevated concentrations of the MaR1 metabolite 14-oxo-MaR1 compared to healthy cells incubated with IL-1β and MaR1 (p = 0.01) (Fig. 4B, Supplemental Tables 1 and 2).

### Diseased tendon cells and tissues show increased PGDH expression

Since tendon stromal cells from diseased donors had an enhanced ability to convert 15-epi-LXA_4_ and MaR1 into metabolites that possess reduced biological actions, we investigated the expression of enzymes implicated in SPM metabolism in healthy and diseased tendon tissues and cells. NAD + dependent 15-Prostaglandin Dehydrogenase (15-PGDH) is a short-chain dehydrogenase/reductase (classified as SDR36C1)^32, 33^, that converts PGE_2_ and LXA_4_ to 15-keto-PGE_2_ and 15-oxo-LXA_4_ respectively^34^. We therefore investigated expression of 15-PGDH mRNA and protein in cells and tissues derived from healthy and diseased human tendons. 15-PGDH mRNA and protein were highly expressed in cells isolated from diseased supraspinatus compared to healthy hamstring tendons after IL-1β treatment for 24 h (p = 0.008) (Fig. 4C and D). Tissues derived from patients with supraspinatus tendinopathy (n = 14) showed increased *15-PGDH* mRNA compared to healthy (subscapularis) shoulder tendons (n = 4) (p = 0.018) (Fig. 4E). Expression of 15-PGDH protein was also determined in these healthy and diseased human shoulder tendon tissues. Immunostaining confirmed increased 15-PGDH protein in diseased compared to healthy shoulder tendons (Fig. 4F). Together these results suggest that the increase in 15-PGDH leads to rapid further metabolism and inactivation of the SPM in cells from diseased patients, thereby blunting their biological actions.

### 15-PGDH inhibition prevents 15-epi-LXA_4_ and MaR1 further conversion

**Figure 5:**
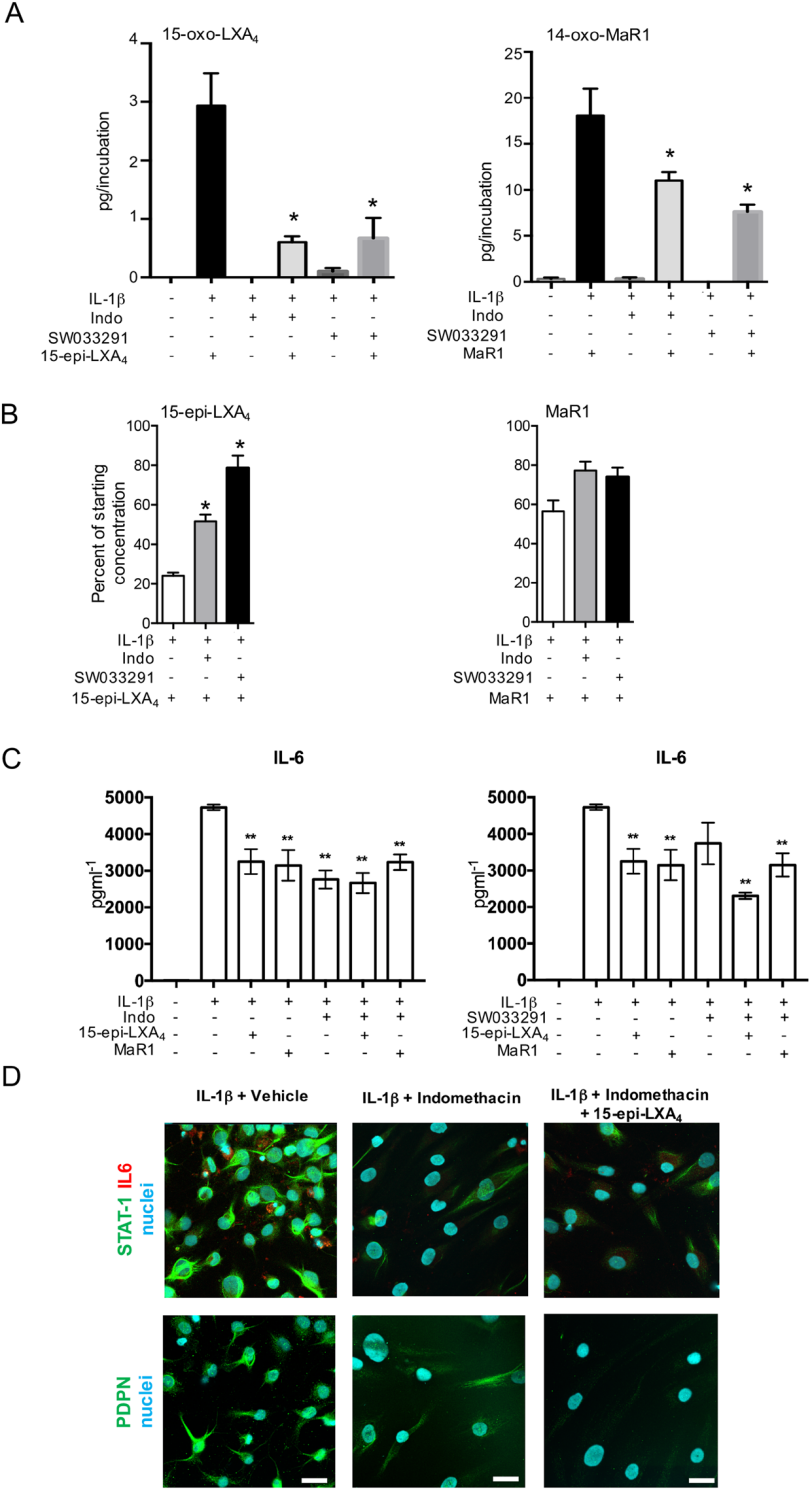
15-PGDH inhibition attenuates the conversion of SPMs to biologically inactive metabolites and regulates markers of inflammation in diseased tendon stromal cells. **(A**,**B**) Diseased tendon stromal cells isolated from patients with supraspinatus tendinopathy were pre-incubated with 10μM indomethacin (Indo) or 25 μM 15-PGDH inhibitor (SW033291) for 2 h, followed by incubation in 10 nM of 15-epi-LXA_4_ or MaR1 or vehicle for 24 h at 37 °C. Incubations were then treated with IL-1β for 24 h at 37 °C. Lipid mediators were identified and quantified using lipid mediator profiling. **(A)** 15-oxo-LXA_4_ and 14-oxo-MaR1 concentrations; (**B**) 15-epi-LXA_4_ and MaR1 concentrations relative to amounts added to each incubation. Statistically significant differences were calculated using one-way ANOVA followed by Tukey post hoc test. Data are shown as means and SEM, n = 3 separate donors. *p < 0.05 vs cells + IL-1β. **(C)** ELISA assay of IL-6 protein secretion from IL-1β stimulated diseased tendon cells in the presence of 15-PGDH inhibitors and 15-epi-LXA_4_ or MaR1. Data are shown as means and SEM, n = 4 separate donors. **P < 0.01. **(D)** Representative immunofluorescence images showing staining for PDPN (green), STAT-1 (green), IL-6 (red) and nuclei (cyan) in IL-1β-stimulated diseased tendon stromal cells incubated in 10 μM indomethacin and 10 nM 15-epi-LXA_4_ or vehicle control media (n = 3 separate donors). Scale bar, 20 μm.

Having identified increased 15-PGDH and enhanced SPM further metabolism in cells from patients with tendinopathy, we investigated whether 15-PGDH was indeed responsible for inactivation of these mediators in cells derived from these patients. For this purpose, we incubated cells with either the 15-PGDH inhibitor SW033291^35^ or indomethacin which in addition to its effects on COX also inhibits 15-PGDH activity^36^, and is used clinically to moderate inflammation^37^. In incubations of IL-1β stimulated diseased tendon stromal cells with either indomethacin or SW033291, we found significantly lower concentrations of 15-oxo-LXA_4_ and 14-oxo-MaR1 levels, and a corresponding increase in the levels of 15-epi-LXA_4_ and MaR1 (Fig. 5A,B Tables 2,3). These results indicated that the elevated 15-PGDH expression was responsible for the blunted actions of these mediators in regulating lipid mediator profiles in stromal cells from patients with tendinopathy.

**Table 2:** SPM profiles in IL-1β stimulated diseased tendon stromal cells incubated in MaR1 and inhibitors of 15-PGDH.

DHA bioactive metabolome	Q1	Q3	Tendon stromal cells Lipid mediators levels (pg/incubation)					
			Disease + IL-1β	Disease + IL-1β + MaR1	Disease + IL-1β + Indo	Disease + IL-1β + MaR1_ + _Indo	Disease + IL-1β + SW033291	Disease + IL-1β + MaR1 + SW033291
RvD1	375	141	0.2 ± 0.2	0.0 ± 0.0	—	—	—	—
RvD2	375	141	0.6 ± 0.4	0.9 ± 0.3	—	—	0.1 ± 0.1	0.2 ± 0.2
RvD3	375	147	0.3 ± 0.3	0.4 ± 0.4	—	—	—	—
RvD4	375	101	1.3 ± 1.3	0.5 ± 0.3	—	—	—	—
RvD5	359	199	34.0 ± 3.8	32.9 ± 7.1	1.3 ± 1.3**	0.3 ± 0.3**	8.6 ± 1.2**	7.1 ± 1.6
RvD6	359	101	14.9 ± 2.5	13.5 ± 2.3	4.1 ± 0.5**	7.4 ± 1.1**	3.1 ± 1.3**	2.0 ± 0.4
17R-RvD1	375	141	1.2 ± 0.7	0.6 ± 0.2	—	0.2 ± 0.2	—	0.1 ± 0.1
17R-RvD3	375	147	2.5 ± 1.5	2.4 ± 0.9	— *	—	—	0.0 ± 0.0
PD1	359	153	7.0 ± 1.1	3.5 ± 0.8*	1.5 ± 1.0 *	0.1 ± 0.1	1.4 ± 0.7**	0.2 ± 0.2
17R-PD1	359	153	0.2 ± 0.1	0.2 ± 0.2	—	0.0 ± 0.0	0.1 ± 0.1	0.6 ± 0.3
10 S,17S-diHDHA	359	153	34.6 ± 5.2	46.8 ± 4.7	0.6 ± 0.6*	1.7 ± 1.7	0.9 ± 0.9 **	0.8 ± 0.8
MaR1	359	221	0.9 ± 0.9	560.3 ± 64.4 **	0.0 ± 0.0	723.4 ± 42.5**^$^	0.7 ± 0.7	662.3 ± 59.5 **##
MaR2	359	191	0.5 ± 0.3	—	0.7 ± 0.3	1.9 ± 0.2*^$^	2.1 ± 0.5*	— #
7 S,14S-diHDHA	359	221	32.0 ± 5.2	34.9 ± 1.4	—	28.8 ± 1.9^$^	—**	—
4 S,14S-diHDHA	359	101	—	—	—	—	—	—
14-oxo-MaR1	357	248	0.3 ± 0.2	18.1 ± 2.9 **	0.3 ± 0.2	11.0 ± 0.9 ** ^$^	0.2 ± 0.2	7.6 ± 0.8 **#
**n-3 DPA bioactive metabolome**								
RvD1_n-3 DPA_	377	143	3.2 ± 1.9	3.4 ± 0.8	0.1 ± 0.1	1.2 ± 0.6	—	0.5 ± 0.5
RvD2_n-3 DPA_	377	261	0.4 ± 0.3	0.3 ± 0.3	—	0.0 ± 0.0	—	—
RvD5_n-3 DPA_	361	263	25.4 ± 1.7	13.4 ± 2.5	— **	0.7 ± 0.7 **	— **	— **
PD1_n-3 DPA_	361	183	0.9 ± 0.5	1.3 ± 0.3	0.4 ± 0.4	1.5 ± 0.1	4.2 ± 1.8	0.7 ± 0.4
10 S,17S-diHDPA	361	183	16.8 ± 1.7	16.7 ± 2.3	5.2 ± 1.3 *	5.1 ± 1.3	3.3 ± 0.5 **	3.5 ± 0.7 **
MaR1_n-3 DPA_	361	249	3.9 ± 2.1	0.5 ± 0.2	0.4 ± 0.4	1.5 ± 1.5	0.1 ± 0.1	0.1 ± 0.1
**EPA bioactive metabolome**								
RvE1	349	195	—	—	—	0.2 ± 0.0 ^$^	—**	0.2 ± 0.1
RvE2	333	199	49.0 ± 11.7	31.8 ± 2.4	18.9 ± 2.0 *	15.8 ± 3.5	23.6 ± 4.0 *	12.5 ± 1.4 *#
RvE3	333	201	0.4 ± 0.3	0.5 ± 0.3	—	—	—	—
**AA bioactive metabolome**								
LXA_4_	351	217	—	—	—	—	—	—
LXB_4_	351	221	1.4 ± 1.4	17.2 ± 17.2	—	3.1 ± 3.1	—	—
5 S,15S-diHETE	335	235	121.8 ± 14.8	750.1 ± 203.5*	—*	—*	—**	—**
15epi-LXA_4_	351	217	—	—	—	—	—	—
15epi-LXB_4_	351	221	35.0 ± 7.3	69.8 ± 12.1*	18.5 ± 3.6*	33.8 ± 5.3^$^	—**	—**
15-oxo-LXA_4_	349	233	—	0.1 ± 0.1	—	—	0.1 ± 0.0	0.1 ± 0.1
LTB_4_	335	195	—	—	—			
5 S,12S-diHETE	335	195	45.6 ± 45.6	36.4 ± 18.3	—	—	—	
PGD_2_	351	189	50.0 ± 4.0	34.0 ± 9.5	0.7 ± 0.7**	0.7 ± 0.7**	1.2 ± 1.2**	1.0 ± 1.0**
PGE_2_	351	189	1655.4 ± 108.7	1275.0 ± 95.0 *	4.6 ± 3.6**	8.9 ± 1.9**	154.5 ± 14.8**	103.0 ± 14.4**#
PGF_2α_	353	193	258.1 ± 26.0	400.7 ± 138.5	—**	**	2.4 ± 2.2**	3.3 ± 1.9**
TxB_2_	369	169	374.8 ± 31.5	554.3 ± 191.6	—**	—**	3.3 ± 3.0**	8.3 ± 0.6**

Tendon stromal cells (60,000 cells per well) were derived from diseased supraspinatus tendons. Cells were incubated for 24 h (37 °C) with indomethacin (indo; 10 µM), SW033291 (25 µM) or vehicle (2 h, 37 °C), then with MaR1 (10 nM) or vehicle (24 h 37 °C) and with IL-1β (37 °C; 24 h). Incubations were quenched using ice-cold methanol containing deuterium labelled internal standards and lipid mediators (LM) were identified and quantified using LM-profiling (see methods for details). Q1, M-H (parent ion) and Q3, diagnostic ion in the MS-MS (daughter ion). Results are expressed as pg/incubation. Mean ± SEM of n = 3 per incubation. *P < 0.05, **P < 0.01 vs Disease + IL1β incubations. ^$^P < 0.05, ^$^^$^P < 0.01 vs Disease + IL1β  + Indo; ^#^P < 0.05, ^##^P < 0.01 vs Disease + IL1β  + SW. The detection limit was ~0.1 pg. -, Below levels found in media alone.

**Table 3:** SPM profiles in IL-1β stimulated diseased tendon stromal cells incubated in 15-epi-LXA_4_ and inhibitors of 15-PGDH.

DHA bioactive metabolome	Q1	Q3	Tendon stromal cells Lipid mediators levels (pg/incubation)					
			Disease + IL-1β	Disease + IL-1β + 15-epi-LXA_4_	Disease + IL-1β + Indo	Disease + IL-1β + 15-epi-LXA_4 + _Indo	Disease + IL-1β + SW033291	Disease + IL-1β + 15-epi-LXA_4 + _SW033291
RvD1	375	141	0.2 ± 0.2	1.0 ± 1.0	—	—	—	—
RvD2	375	141	0.6 ± 0.4	1.3 ± 0.3	—	—	0.1 ± 0.1	—
RvD3	375	147	0.3 ± 0.3	0.2 ± 0.1	—	—	—	—
RvD4	375	101	1.3 ± 1.3	1.9 ± 0.2	—	—	—	—
RvD5	359	199	34.0 ± 3.8	27.5 ± 5.2	1.3 ± 1.3**	0.3 ± 0.3**	8.6 ± 1.2**	2.1 ± 1.2**
RvD6	359	101	14.9 ± 2.5	20.4 ± 0.4*	4.1 ± 0.5**	4.1 ± 0.2**	3.1 ± 1.3**	1.0 ± 0.7**
17R-RvD1	375	141	1.2 ± 0.7	2.4 ± 1.3	—	0.3 ± 0.3	—	—
17R-RvD3	375	147	2.5 ± 1.5	4.5 ± 1.1	— *	—	—	—
PD1	359	153	7.0 ± 1.1	7.0 ± 1.0	1.5 ± 1.0*	0.8 ± 0.7**	1.4 ± 0.7**	0.7 ± 0.7**
17R-PD1	359	153	0.2 ± 0.1	0.3 ± 0.3	—	0.0 ± 0.0	0.1 ± 0.1	0.3 ± 0.2
10 S,17S-diHDHA	359	153	34.6 ± 5.2	64.3 ± 6.2*	0.6 ± 0.6*	1.8 ± 1.8**	0.9 ± 0.9**	—**
MaR1	359	221	0.9 ± 0.9	13.2 ± 2.1**	0.0 ± 0.0	1.2 ± 1.2	0.7 ± 0.7	—
MaR2	359	191	0.5 ± 0.3	1.0 ± 0.1	0.7 ± 0.3	1.0 ± 0.6	2.1 ± 0.5*	—
7 S,14S-diHDHA	359	221	32.0 ± 5.2	22.1 ± 6.7	—	0.4 ± 0.4**	—**	—**
4 S,14S-diHDHA	359	101	—	—	—	—	—	—
14-oxo-MaR1	357	248	0.3 ± 0.2	0.4 ± 0.4	0.3 ± 0.2	0.1 ± 0.1	0.2 ± 0.2	—
**n-3 DPA bioactive metabolome**								
RvD1_n-3 DPA_	377	143	3.2 ± 1.9	4.7 ± 0.6	0.1 ± 0.1	0.2 ± 0.2	—	—
RvD2_n-3 DPA_	377	261	0.4 ± 0.3	—	—	0.0 ± 0.0	—	—
RvD5_n-3 DPA_	361	263	25.4 ± 1.7	14.6 ± 2.4 *	— **	1.2 ± 1.2	— **	— **
PD1_n-3 DPA_	361	183	0.9 ± 0.5	2.2 ± 1.1	0.4 ± 0.4	0.8 ± 0.2	4.2 ± 1.8	0.9 ± 0.3
10 S,17S-diHDPA	361	183	16.8 ± 1.7	16.8 ± 2.3	5.2 ± 1.3 *	6.0 ± 0.7 **	3.3 ± 0.5 **	3.3 ± 1.0 **
MaR1_n-3 DPA_	361	249	3.9 ± 2.1	2.8 ± 0.6	0.4 ± 0.4	0.4 ± 0.2	0.1 ± 0.1	—
**EPA bioactive metabolome**								
RvE1	349	195	—	—	—	0.0 ± 0.0	— **	0.1 ± 0.1
RvE2	333	199	49.0 ± 11.7	44.3 ± 9.4	18.9 ± 2.0 *	22.3 ± 4.6 *	23.6 ± 4.0 *	24.6 ± 4.0
RvE3	333	201	0.4 ± 0.3	0.0 ± 0.0	—	0.0 ± 0.0	—	—
**AA bioactive metabolome**								
LXA_4_	351	217	—	15.9 ± 10.6	—	4.9 ± 0.5** $	—	2.4 ± 1.4
LXB_4_	351	221	1.4 ± 1.4	—	—	0.0 ± 0.0	—	—
5 S,15S-diHETE	335	235	121.8 ± 14.8	770.0 ± 168.4**	— *	2.9 ± 2.9*	— **	—
15epi-LXA_4_	351	217	—	253.2 ± 16.3**	—	291 ± 20.2^**$$^	—	297.1 ± 23.9^**##^
15epi-LXB_4_	351	221	35.0 ± 7.3	45.5 ± 6.6	18.5 ± 3.6*	21.3 ± 5.9	— **	5.4 ± 2.7*
15-oxo-LXA_4_	349	233	—	3.0 ± 0.5*	—	0.6 ± 0.1^$$^	0.1 ± 0.0	0.5 ± 0.2^* #^
LTB_4_	335	195	—		—			
5 S,12S-diHETE	335	195	45.6 ± 45.6	40.9 ± 31.1	—	0.0 ± 0.0	—	—
PGD_2_	351	189	50.0 ± 4.0	61.0 ± 9.4	0.7 ± 0.7**	30.8 ± 3.8^* #^	1.2 ± 1.2 **	29.4 ± 6.0^*#^
PGE_2_	351	189	1655.4 ± 108.7	1275.0 ± 95.0 **	4.6 ± 3.6**	4.8 ± 3.8**	154.5 ± 14.8**	74.6 ± 20.7** #
PGF_2α_	353	193	258.1 ± 26.0	258.8 ± 51.3	—**	0.0 ± 0.0**	2.4 ± 2.2**	5.7 ± 5.3**
TxB_2_	369	169	374.8 ± 31.5	367.9 ± 77.2	— **	0.0 ± 0.0**	3.3 ± 3.0**	7.9 ± 7.4**

Tendon stromal cells (60,000 cells per well) were derived from diseased supraspinatus tendons. Cells were incubated for 24 h (37 °C) with indomethacin (indo; 10 µM), SW033291 (25 µM) or vehicle (2 h, 37 °C), then with 15-epi-LXA_4_ (10 nM) or vehicle (24 h 37 °C) and with IL-1β (37 °C; 24 h). Incubations were quenched using ice-cold methanol containing deuterium labelled internal standards and lipid mediators (LM) were identified and quantified using LM-profiling (see methods for details). Q1, M-H (parent ion) and Q3, diagnostic ion in the MS-MS (daughter ion). Results are expressed as pg/incubation. Mean ± SEM of n = 3 per incubation. *P < 0.05, **P < 0.01 vs Disease + IL1β incubations. ^$^P < 0.05, ^$$^P < 0.01 vs Disease + IL1β  + Indo; ^#^P < 0.05, ^##^P < 0.01 vs Disease + IL1β  + SW. The detection limit was ~ 0.1 pg. -, Below levels found in media alone.

We therefore next questioned whether incubating cells with both an SPM and indomethacin or SW033291 would have additive actions on regulating markers of inflammation in tendon stromal cells. Assessment of IL-6 production in these cells demonstrated that whereas there were no additive actions with indomethacin, incubation of SW033291 together with 15-epi-LXA_4_ displayed additive actions in down-regulating the concentrations of this inflammatory cytokine (Fig. 5C). We also found that co-incubation of 15-epi-LXA_4_ with indomethacin displayed additive actions in regulating PDPN and STAT-1 expression (Fig. 5D) compared to incubation in 15-epi-LXA_4_ alone (Fig. 2E and G).

## Discussion

Specialized proresolving mediators (SPM) including lipoxins, resolvins, protectins and maresins initiate the highly active and coordinated process of resolution^38^, regulating the duration and magnitude of inflammation and promoting restoration of tissue homeostasis after infection and/or injury^14, 16, 39^. Whilst SPMs are implicated in resolving acute inflammation via cells of the innate immune system, these bioactive mediators are also associated with chronic inflammatory diseases^17, 18^. Tissue-resident stromal cells such as fibroblasts are emerging as an important cell type implicated in mediating the resolution of inflammation in wound healing, periodontal disease, pulmonary inflammation and Rheumatoid Arthritis^24, 25, 40, 41^. Stromal fibroblasts actively participate in inflammatory responses and are implicated in governing the persistence of inflammatory disease through a variety of mechanisms including stromal fibroblast activation, recruitment and retention of immune cells, inhibition of leucocyte apoptosis and immunological memory^13^.

Chronic inflammation is a common feature of musculoskeletal soft tissue diseases including tendinopathy^9^. Tendons possess a low rate of tissue turnover^42^, therefore damage accumulated may be long lasting as diseased tissue heals by fibrosis and not regeneration. Current therapeutic strategies focus on ameliorating the pain associated with disease but do not address the underlying biological mechanisms underpinning the development of chronic inflammation. Resolution of inflammation has not been well studied in the context of diseased human musculoskeletal soft tissues.

In the present study we found that incubation of tendon-derived stromal cells with IL-1β up regulated the production of pro-inflammatory eicosanoids and proresolving SPM. Furthermore, the production of select pro-resolving mediators was significantly higher in tendon stromal cells from patients with tendinopathy compared with those from healthy volunteers. These results are in line with an increased SPM biosynthetic enzyme observed in these cells, pointing to a status of dysregulated resolution, where in response to an inflammatory stimulus, in this case IL-1β, the up regulation of tissue protective mediators is not sufficient to counter regulate the inflammatory profile. Of note, addition of either 15-epi-LXA_4_ or MaR1 to the cell incubations also led to a feed forward production in endogenous SPM production by both healthy volunteer and diseased tendon stromal cells. These findings are also in line with an increased expression of the SPM biosynthetic enzyme ALOX15 observed in these cells as well as with published findings in other experimental systems including peritonitis^43^ and asthma^44^, where the administration of one SPM triggers the formation of different SPM that contribute to the resolution of inflammation. These findings suggest that diseased tendon cells display a pro-inflammatory and dysregulated resolution profile as summarized in Figure S4. Our findings are consistent with earlier reports that demonstrate pro-inflammatory mediators including IL-1β induce the production of PGE_2_ in cultures of tendon cells^45, 46^.

Having observed that a select group of pro-resolving mediators was up regulated in patient-derived compared to healthy tendon stromal cells, we queried whether these autacoids carried biological actions in regulating molecular aspects of tendon inflammation. Indeed 15-epi-LXA_4_ and MaR1 regulated lipid mediator production in both healthy and patient tendon-derived stromal cells. In addition, incubation of patient derived tendon cells with these SPM also led to a reduction of IL-6, STAT-1 and PDPN, that we have previously found to be associated with disease severity^9, 11^. Of note, we found that the biological actions of both 15-epi-LXA_4_ and MaR1 were blunted in patient-derived tendon cells, this decreased effectiveness was associated with an increased further metabolism of these mediators to their inactive metabolites. This process of inactivation was at least in part reliant on 15-PGDH since this enzyme was found to be up regulated in patient cells compared with tendon stromal cells from healthy volunteers. Furthermore, inhibition of this enzyme using either indomethacin or SW033291 led to increased recovery of both 15-epi-LXA_4_ and MaR1 and a reduction in the further metabolism of these mediators in diseased tendon stromal cell incubations. 15-PGDH has been recently shown to negatively regulate tissue repair and regeneration in murine models of bone marrow, colon and liver injury^35^. Zhang *et al*., illustrated that 15-PGDH blockade potentiated repair in multiple murine tissues without apparent adverse effects. Having found that the 15-PGDH inhibitors reduced both SPM further metabolism and prostaglandin production, we also queried whether co-incubation of either 15-epi-LXA_4_ or MaR1 with these inhibitors moderated the pro-inflammatory phenotype of diseased tendon stromal cells. In these incubations we found a further reduction in the expression of IL-6, STAT-1 and PDPN as well as prostaglandins, although this was not statistically significant. Future experiments will need to investigate the potential of this approach and the possibility of obtaining additive or even synergistic actions with a dual pronged approach in controlling soft tissue inflammation, in line with actions observed in bacterial infections where SPMs lower the required doses of antibiotics required to clear infections^47^.

We acknowledge there are potential limitations with the use of hamstring tendon as a comparator to diseased tendons including tendon type and donor age differences. However, hamstring tendon was taken from live healthy donors without history of tendinopathy. We believe this is a more suitable comparator than cadaveric shoulder tendon tissues where little is known about whether the tendons were healthy or diseased and tendons were not affected by post mortem changes.

The findings from this study suggest that tendinopathy is characterized by a status of dysregulated resolution that results from an up regulation of 15-PGDH leading to the rapid inactivation of SPM. Incubation of tendon-derived stromal cells from both healthy volunteers and patients with tendinopathy in SPM including 15-epi-LXA_4_ or MaR1 induced further release of proresolving mediators and counter regulated the expression of pro-inflammatory molecules including PGE_2_, IL-6, STAT-1 and PDPN. In addition, our findings suggest that a dual pronged approach, using pro-resolving mediators together with inhibitors to 15-PGDH may represent a novel therapeutic strategy to reduce local inflammation and promote tissue repair and regeneration.

## Materials and Methods

### Study Approval

Healthy and diseased tendon tissues were collected under research ethics from the Oxford Musculoskeletal Biobank (09/H0606/11). Full informed consent according to the Declaration of Helsinki was obtained from all patients. Experimental protocols were performed at the University of Oxford in accordance with research ethics from the Oxford Musculoskeletal Biobank (09/H0606/11).

### Collection of human tendon tissues

All patients were recruited from orthopedic referral clinics where the structural integrity of the supraspinatus tendon was determined ultrasonographically. Patients presenting to the referral shoulder clinic had failed non-operative treatment, including a course of physical therapy, and had experienced pain for a minimum of 3 months. Patients completed the Oxford Shoulder Score (OSS), a validated and widely used clinical outcome measure scoring from 0 (severe pathology) to 48 (normal function). Diseased tendon tissues (supraspinatus tendon tears) were collected at the time of surgical debridement of the edges of the torn tendons from 28 male and female patients aged between 44 and 75 (mean 55 ± 18.3 years). All patients were symptomatic and had small to medium tears (≤1 cm to ≤3 cm in anterior-posterior length). Exclusion criteria for all patients in this study included previous shoulder surgery, other shoulder pathology, rheumatoid arthritis and systemic inflammatory disease. Samples of healthy supraspinatus (n = 5) and subscapularis tendons (n = 4) were collected intra-operatively from male and female patients between 25–65 years of age that were undergoing shoulder surgery for post-traumatic instability. Healthy hamstring tendons were collected from 15 male and female patients undergoing surgical reconstruction of their anterior cruciate ligament. All patients were aged between 18 and 48 (mean 25.2 ± 11 years).

### Isolation of tendon-derived stromal cells from healthy and diseased tendons

Fresh samples of healthy hamstring or diseased supraspinatus tendons were cut into 2mm^3^ explants and incubated in DMEM F12 media (Lonza) containing 50% fetal calf serum (FCS, Labtech) and 1% Penicillin Streptomycin (Pen-Strep, Lonza). Media were replaced every 4 days and cells allowed to grow out from explants over time in a tissue culture incubator at 37 **°**C and 5% CO_2_. Once cells were 90% confluent, explants were removed and media replaced with DMEM F12 containing 10% FCS and 1% Pen-Strep. Healthy and diseased tendon cells between passages 1–3 were used for all experiments. Our previous characterization of human tendon derived stromal cells demonstrated cells used for *in vitro* experiments were CD45^neg^ and CD34^neg^. Diseased tendon cells are known to highly express markers of stromal fibroblast activation including PDPN, CD106 and CD248 compared to healthy tendon derived cells^11^.

### Treatment of tendon-derived stromal cells with IL-1β for bioactive lipid mediator profiling

As IL-1β induces NF-κB target genes known to be highly expressed in early stage tendinopathy^9^, we investigated bioactive pro-resolving lipid mediator profiles in cells derived from healthy and diseased human tendons in the presence of IL-1β. Tendon-derived stromal cells from healthy hamstring (n = 8) and diseased supraspinatus tendons (n = 8) were seeded at a density of 60,000 cells per well in a 6 well plate. Tendon cells were allowed to reach 80% confluence prior to stimulation with IL-1β (Merck, 10ngml^—1^) in DMEM F12 phenol red free medium (Gibco) containing 1% heat inactivated human serum (Sigma) and 1% penicillin-streptomycin. Non-treated cells (vehicle only, containing 0.1% endotoxin free BSA, Sigma) served as controls for each experiment. After treatment, cells were then incubated at 37 **°**C and 5% CO_2_ until harvest of the media and lysate for bioactive lipid mediator profiling after 24 h.

### Modulating bioactive lipid mediator profiles with 15-epi-LXA_4_ or MaR1 in IL-1β stimulated tendon-derived stromal cells

Tendon stromal cells were derived from healthy hamstring (n = 7) and diseased supraspinatus tendons (n = 6). Cells were seeded at a density of 60,000 cells per well. Once cells were at 80% confluence, they were pre-incubated with 0.1 nM or 10 nM 15-epi-LXA_4_ (Cayman Chemical) or 10 nM MaR1 (Cayman Chemical) for 24 h in DMEM F12 phenol red free medium (Gibco) containing 1% heat inactivated human serum (Sigma) and 1% penicillin-streptomycin. Cells were subsequently stimulated with IL-1β (10ngml^—1^) as described above in the presence of media containing either 0.1 nM or 10 nM 15-epi-LXA_4_, 10 nM MaR1 or vehicle only control media. After IL-1β treatment, cells were shielded from light and incubated at 37°C and 5% CO_2_ until harvest of the media and lysate for bioactive lipid mediator profiling after 24 h. Parallel experiments were conducted and cell lysates harvested to investigate if incubating cells in 15-epi-LXA_4_ or MaR1 modulated expression of pro-inflammatory genes expressed by diseased tendon stromal cells. The concentration and integrity of mediators used for these incubations were validated using UV-spectrophotometry and LC-MS-MS in accordance with published criteria^25^.

### Bioactive lipid mediator profiling of IL-1β stimulated healthy and diseased tendon stromal cells

Media and lysate samples were stored at −80 °C prior to analysis. 2 volumes of ice cold MeOH containing deuterated internal standards (d_4_-LTB_4_, d_8_-5S-HETE, d_4_-PGE_2_, d_5_-LXA_4_ and d_5_-RvD2, 500 pg each) were added to the samples. These were then kept at −20 °C for 45 minutes to allow for protein precipitation and subjected to solid phase extraction as per previous publication^26^. Methyl formate fractions were then brought to dryness using a TurboVap LP (Biotage) and products suspended in water-methanol (50:50 vol:vol) for LC-MS-MS. A Shimadzu LC-20AD HPLC and a Shimadzu SIL-20AC autoinjector (Shimadzu, Kyoto, Japan), paired with a QTrap 5500 (ABSciex, Warrington, UK) were utilised and operated as described^26^. To monitor each lipid mediator and respective pathways, a Multiple Reaction Monitoring (MRM) method was developed with diagnostic ion fragments and identification using recently published criteria^26^, including matching retention time (RT) to synthetic and authentic materials and at least six diagnostic ions for each lipid mediator. Calibration curves were obtained for each using authentic compound mixtures and deuterium labeled lipid mediator at 0.78, 1.56, 3.12, 6.25, 12.5, 25, 50, 100, and 200 pg. Linear calibration curves were obtained for each lipid mediator, which gave r^2^ values of 0.98–0.99.

### *15-PGDH* mRNA expression in healthy and diseased tendon tissues and cells

Tendon-derived stromal cells from healthy hamstring (n = 5) and diseased supraspinatus tendons (n = 5) were seeded at a density of 15,000 cells per well in a 24 well plate. Tendon cells were allowed to reach 80% confluence prior to stimulation with IL-1β (Merck, 10ngml^—1^) in DMEM F12 phenol red free medium (Gibco) containing 1% heat inactivated human serum (Sigma) and 1% penicillin-streptomycin. Non-treated cells (vehicle only, containing 0.1% endotoxin free BSA, Sigma) served as controls for each experiment. After cytokine treatment, cells were then incubated at 37 °C and 5% CO_2_ until harvest of the cell lysate for mRNA after 24 h. For tissues, samples of healthy subscapularis (n = 4) and diseased supraspinatus tendons (n = 14) were snap frozen and stored at −80 °C. RNA isolation, cDNA synthesis and quantitative PCR were performed using previously published protocols^9^. Pre-validated Qiagen primer assays (*15-PGDH*, *STAT-1*, *IL6*, *PDPN*, *β-actin* and *GAPDH*) were used for qPCR. Results were calculated using the ddCt method using reference genes for human *β-actin* and *GAPDH*. Results were consistent using these reference genes and data are shown normalized to *β-actin*.

### Immunofluorescence for 15-PGDH in healthy and diseased tendons

Tendon samples were immersed in 10% buffered formalin, processed using a Leica ASP300S tissue processor and embedded in paraffin wax. Tissues were sectioned to 4μm onto adhesive glass slides. For antigen retrieval, slides were baked at 60 °C for 60 minutes and tissue sections were taken through deparaffinisation and target retrieval steps (high pH heat mediated antigen retrieval) using an automated PT Link (Dako). Immunofluorescence staining protocols and image acquisition are adapted from a previously published protocol^9^. Sections were incubated with the primary antibody anti-15-PGDH (Abcam, ab118185). For negative controls the primary antibody was substituted for universal isotype control antibodies: cocktail of mouse IgG_1_, IgG_2a_, IgG_2_b, IgG_3_ and IgM (Dako) (Figure S5).

### Immunocytochemistry for healthy and diseased tendon stromal cells

Tendon stromal cells were grown in chamber slides and treated as above. Cells were fixed in ice cold methanol for 5 mins and washed twice in PBS. Immunofluorescence staining protocols and image acquisition are adapted from a previously published protocol^9^. Tendon stromal cells isolated from 3 healthy hamstring donors and 3 patients with supraspinatus tendinopathy were incubated with the following primary antibodies: anti-ALOX15 (Abcam ab119774), anti-15-PGDH (Abcam, ab118185), anti-STAT-1 (Abcam ab29045), anti-COX2 (Abcam ab15191), anti-Podoplanin (Abcam ab10288) and anti-IL-6 (Abcam 9324) in PBS containing 5% goat serum in Saponin for 3 hrs at room temperature. For negative controls the primary antibody was substituted for universal isotype control antibodies: cocktail of mouse IgG_1_, IgG_2a_, IgG_2_b, IgG_3_ and IgM (Dako) and rabbit immunoglobulin fraction of serum from non-immunized rabbits, solid phase absorbed. Isotype control staining is shown in Figure S5.

### Immunofluorescence imaging

Immunofluorescence images were acquired on a Zeiss LSM 710 confocal microscope using a × 40 oil immersion objective (NA = 1.3). The fluorophores of POPO-1, Alexa Fluor 488, Alexa Fluor 568, and Alexa Fluor 633 were excited using the 405 nm, 488 nm, 561 nm, and 633 nm laser lines respectively. To minimize bleed-through, all channels were acquired sequentially. Averaging was set to 2 and the pinhole was set to approximately 1 Airy unit. Two-dimensional image reconstructions were created using ZEN 2009 (Zeiss).

### Treatment of diseased tendon stromal cells with inhibitors of 15-PGDH

Tendon stromal cells were derived from diseased supraspinatus tendons. Cells were seeded at a density of 60,000 cells per well in 6 well plates for SPM profiles, (6 donors). Once cells were at 80% confluence, they were pre-incubated in 10μM indomethacin (Sigma) or 25μM 15-PGDH inhibitor (SW033291, Tocris) in DMEM F12 phenol red free medium containing 1% heat inactivated human serum and 1% penicillin-streptomycin. After 2 h, 10 nM 15-epi Lipoxin A_4_ (Cayman Chemical) or 10 nM Maresin 1 (Cayman Chemical) were added and cells further incubated for 24 h. Healthy and diseased cells were then stimulated with IL-1β (10ngml^—1^), non-treated (vehicle only) IL-1β stimulated cells served as controls for each experiment. Cells were shielded from light and incubated at 37 °C and 5% CO_2_ until harvest of the media and lysate for bioactive lipid mediator profiling 24 h after stimulation with IL-1β.

### Quantification of Interleukin-6 in tissue culture media from diseased tendon stromal cells

IL-6 is an important cytokine implicated in tissue inflammation. IL-6 in tissue culture supernatants was measured using enzyme-linked immunosorbent assay (ELISA) reagents (BD Biosciences). Minimum detectable IL-6 concentration for this assay was 2.2 pgml^—1^. Optical density was read on a spectrophotometric ELISA plate reader (FLUOstar Omega, BMG Labtech) and analysed using MARS data analysis software.

### Statistics

Statistical analyses were performed using GraphPad Prism 7 (GraphPad Software). Normality was tested using the Shapiro-Wilk normality test. Pairwise Mann Whitney U tests were used to test for differences between mRNA expression of *ALOX12*, *ALOX15*, *PTGS2*, *IL6*, *STAT-1*, *PDPN* and *15-PGDH* in IL-1β treated tendon stromal cells in the presence or absence of SPM and inhibitors of 15-PGDH. Pairwise Mann Whitney U tests were used to test for differences between IL-6 protein secretion in IL-1β treated tendon stromal cells in the presence or absence of SPM and inhibitors of 15-PGDH. Pairwise Mann Whitney U tests were used to test for differences between *15-PGDH* mRNA expression in healthy and diseased tendon tissues and cells. Analysis of bioactive lipid mediator profiles from healthy and diseased tendon cells was performed using multivariate statistical analysis, orthogonal-partial least squares-discriminant analysis (o-PLS-DA) was performed using SIMCA 14.1 software (Umetrics, Umea, Sweden) following unit variance scaling of LM amounts. PLS-DA is based on a linear multivariate model that identifies variables that contribute to class separation of observations (cell incubations) on the basis of their variables (LM levels). During LM classification, observations were projected onto their respective class model. The score plot illustrates the systematic clusters among the observations (closer plots presenting higher similarity in the data matrix). Loading plot interpretation identified the variables with the best discriminatory power (Variable Importance in Projection greater then 1) that were associated with tight clusters for lipid mediator profiles obtained from incubations with cells from healthy volunteers or patients with tendinopathy. Data are shown as mean and SEM, where n is the biological replicate (human donor of cells derived from healthy or diseased tendons). *P* < 0.05 was considered statistically significant.

### Data Availability

All data generated from this study are included in this published article and its Supplementary Information files.

## Electronic supplementary material

Supplementary Information
