# Supplementary material for: Increased 15-PGDH expression leads to dysregulated resolution responses in stromal cells from patients with chronic tendinopathy
Source: Sci Rep. 2017 Sep 8;7:11009. doi: 10.1038/s41598-017-11188-y (PMC5591234; doi:10.1038/s41598-017-11188-y)
Supplement: Supplementary file 1 — Supplementary Information [file 41598_2017_11188_MOESM1_ESM.pdf]

Supplementary Information

Increased 15-PGDH expression leads to dysregulated resolution responses in stromal cells from patients with chronic tendinopathy

Stephanie G Dakin*[1], Lucy Ly[2], Romain A. Colas[2], Udo Oppermann[1,3], Kim Wheway[1], Bridget Watkins[1], Jesmond Dalli*[2+], Andrew J Carr[1+].

Supplementary Figures:

Figure S1. Tendon stromal cell lipid mediator profiles.
Figure S2. 15-epi-LXA_4 up regulates SPM levels and down regulates prostaglandin production in healthy and diseased tendon stromal cells.
Figure S3. MaR1 down regulates prostaglandin production in healthy and diseased tendon stromal cells.
Figure S4. Schematic summarising dysregulated resolution responses in diseased tendon stromal cells and proposed therapeutic strategy to potentiate the bioactivity of proresolving mediators.
Figure S5. Isotype control staining of tissues and cells isolated from diseased human tendons.

Supplementary Tables:

Supplement Table 1. 15-epi-LXA_4 and MaR1 up regulate SPM and reduce inflammation-initiating eicosanoids in healthy tendon stromal cells.
Supplement Table 2. Regulation of lipid mediator profiles by 15-epi-LXA_4 and MaR1 in diseased tendon stromal cells.

Figure S1

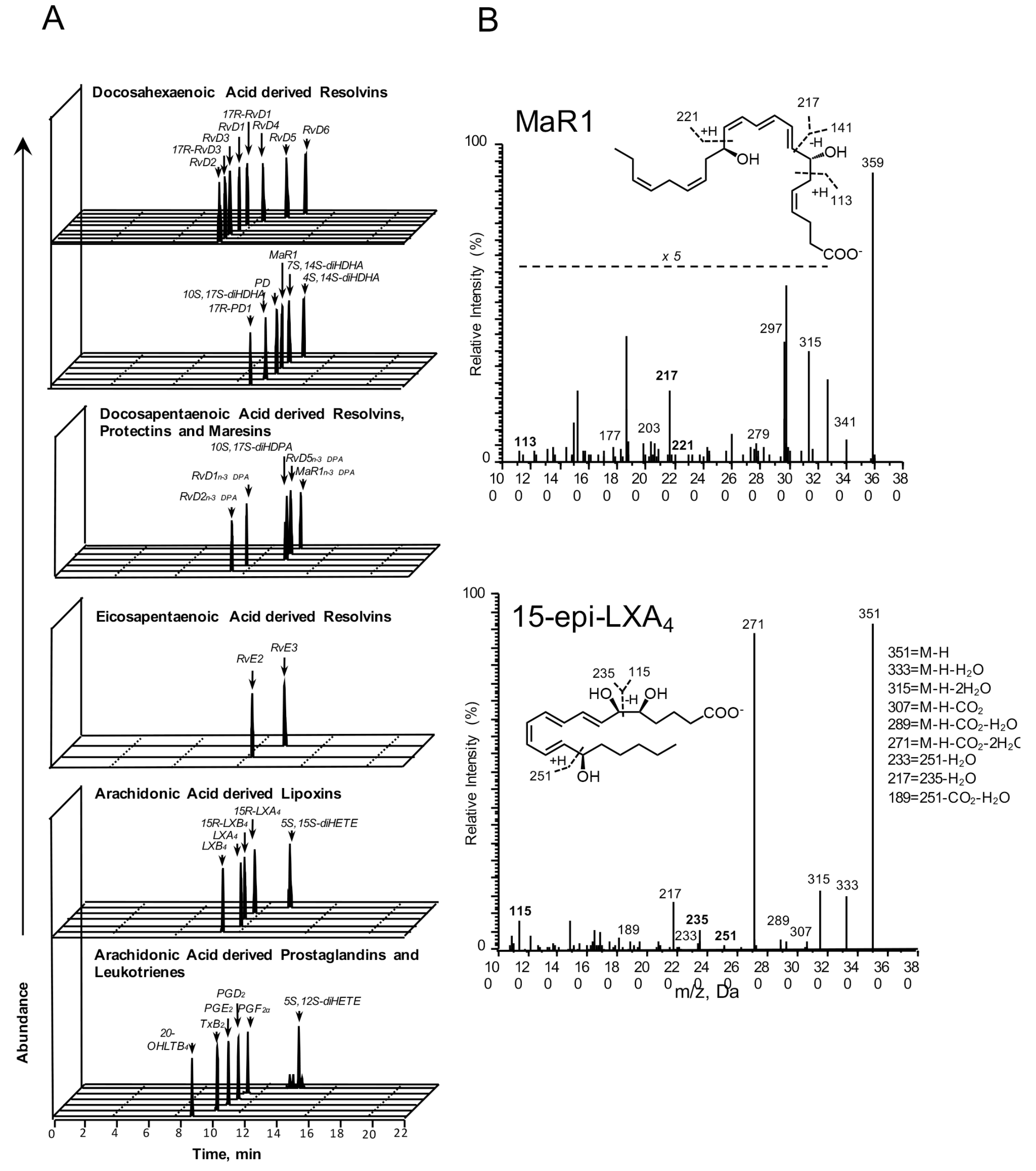

Figure S1. Tendon stromal cell lipid mediator profiles. Tendon stromal cells (60,000 cells per well) were derived from healthy hamstring (n=8 donors) or diseased supraspinatus tendons (n=8 donors). Cells were cultured in DMEM F12 phenol red free medium containing 1% heat inactivated human serum to 80% confluence and incubated with IL-1β for 24h. Media and cells were harvested and placed in ice-cold methanol containing deuterium labelled internal standards. LM were then extracted and profiled. **(A)** Representative MRM chromatograms for lipid mediators identified in healthy and disease cells. **(B)** MS-MS fragmentation spectra employed in the identification of MaR1 and 15-epi-LXA_4. Results are representative of n=16 donors.

Figure S2

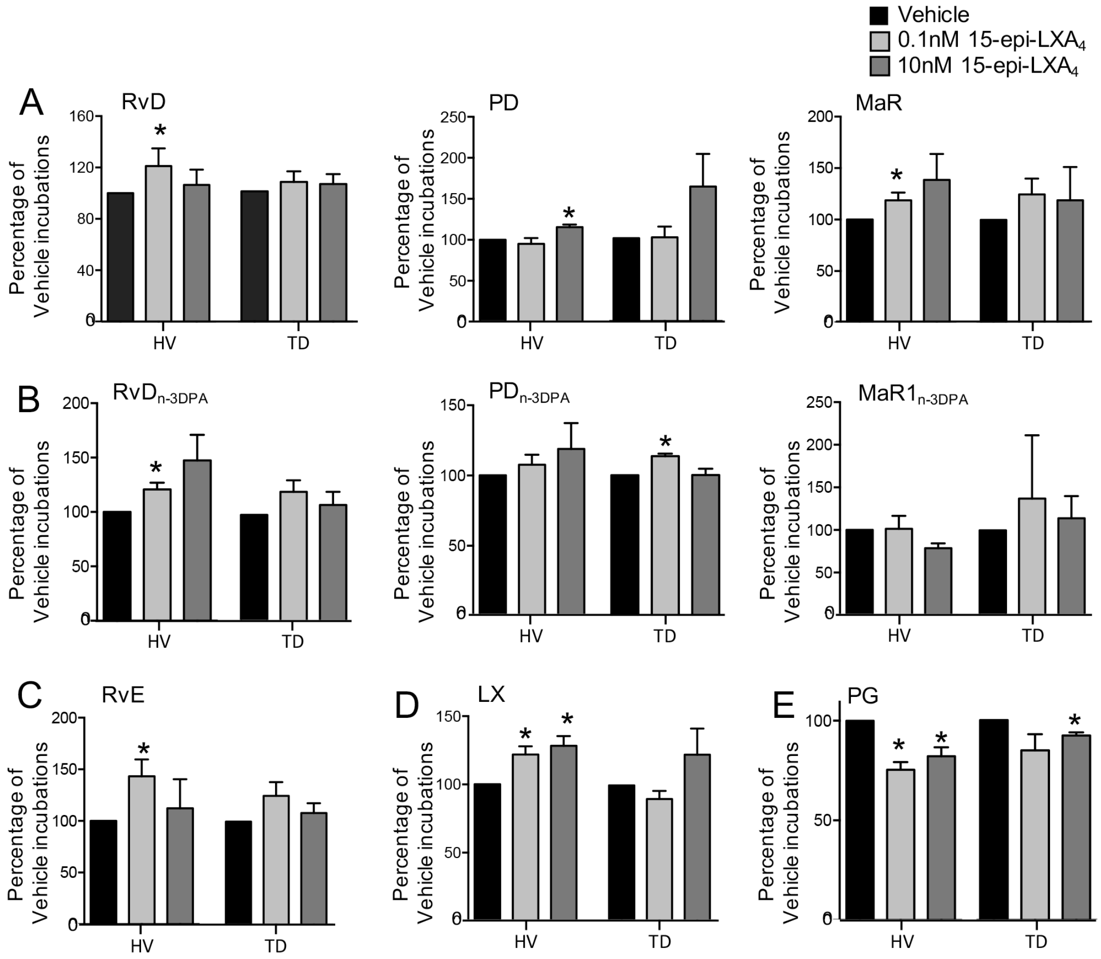

Figure S2. 15-epi-LXA_4 up regulates SPM levels and down regulates prostaglandin production in healthy and diseased tendon stromal cells. Tendon stromal cells were derived from healthy hamstring (H, n=7 donors) or diseased supraspinatus tendons (TD, n=6 donors). Cells were incubated with 15-epi-LXA_4 (0.1 or 10nM) or Vehicle for 24h at 37°C then with IL-1β (10ngml^{-1}) for 24h. LM were identified and quantified using LM profiling (see methods for details). **(A)** DHA-derived RvD, PD, MaR **(B)** n-3 DPA-derived $RvD_{n\text{-}3\ DPA}$, $PD_{n\text{-}3\ DPA}$, $MaR1_{n\text{-}3\ DPA}$, **(C)** EPA-derived RvE **(D)** AA-derived (LX) and **(E)** PG in tendon stromal cells from healthy volunteers (HV) and patients with tendinopathy (TD). Results are mean ± SEM n=7 HV and 6 TD per group. *P < 0.05 vs respective vehicle group.

Figure S3

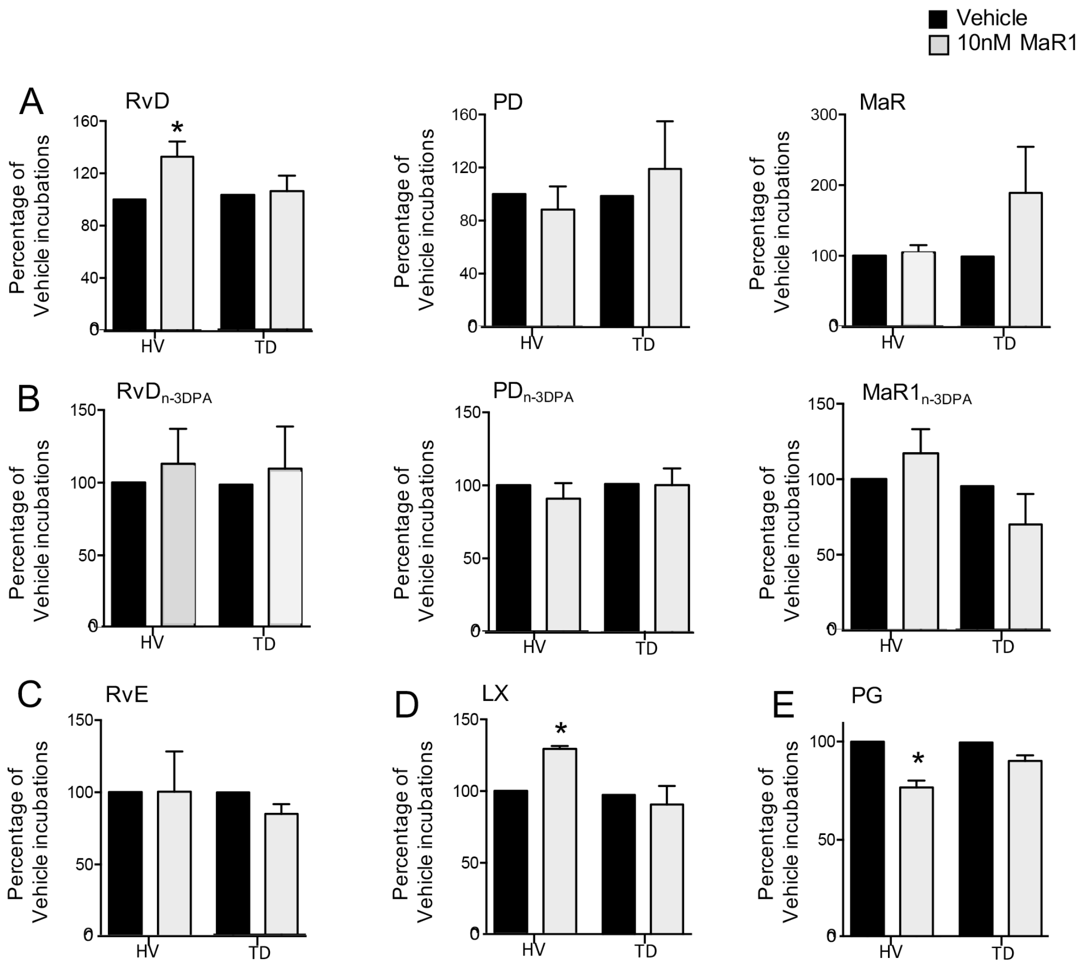

Figure S3. MaR1 down regulates prostaglandin production in healthy and diseased tendon stromal cells. Tendon stromal cells were derived from healthy hamstring (H, n=7 donors) or diseased supraspinatus tendons (TD, n=6 donors). Cells were incubated with MaR1 (10nM) or Vehicle for 24h at 37°C then with IL-1β (10ngml^{-1}) for 24h. LM were identified and quantified using LM profiling (see methods for details). **(A)** DHA-derived RvD, PD, MaR **(B)** n-3 DPA-derived $RvD_{n\text{-}3\ DPA}$, $PD_{n\text{-}3\ DPA}$, $MaR1_{n\text{-}3\ DPA}$, **(C)** EPA-derived RvE **(D)** AA-derived (LX) and **(E)** PG in tendon stromal cells from healthy volunteers (HV) and patients with tendinopathy (TD). Results are mean ± SEM n=7 HV and 6 TD per group. *P < 0.05 vs respective vehicle group.

Figure S4

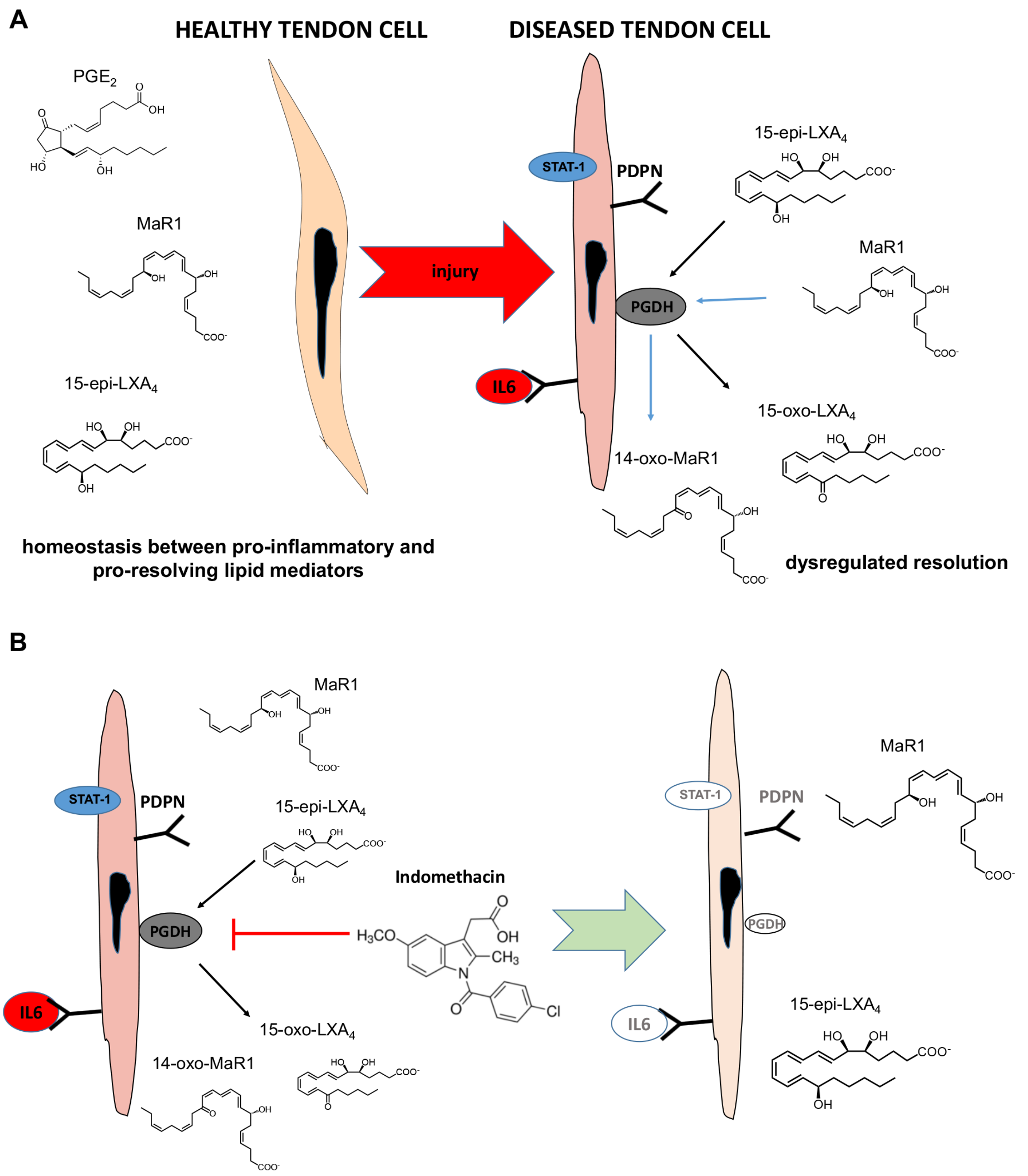

Figure S4. Schematic summarising dysregulated resolution responses in diseased tendon stromal cells and proposed therapeutic strategy to potentiate the bioactivity of proresolving mediators. (A) Stromal cells derived from patients with tendinopathy show rounded morphology, exhibit a pro-inflammatory profile and highly express STAT-1, PDPN, IL-6 and 15-PGDH compared to tendon cells from healthy donors. Diseased tendon stromal cells show an enhanced capacity to convert 15-epi-LXA$_4$ and MaR1 into respective oxo-metabolites with reduced bioactivity, mediated via 15-PGDH metabolism. **(B)** Co-treatment with indomethacin potentiates the bioactions of proresolving mediators including 15-epi-LXA$_4$ and MaR1 and modulates the pro-inflammatory phenotype of diseased tendon stromal cells.

Figure S5

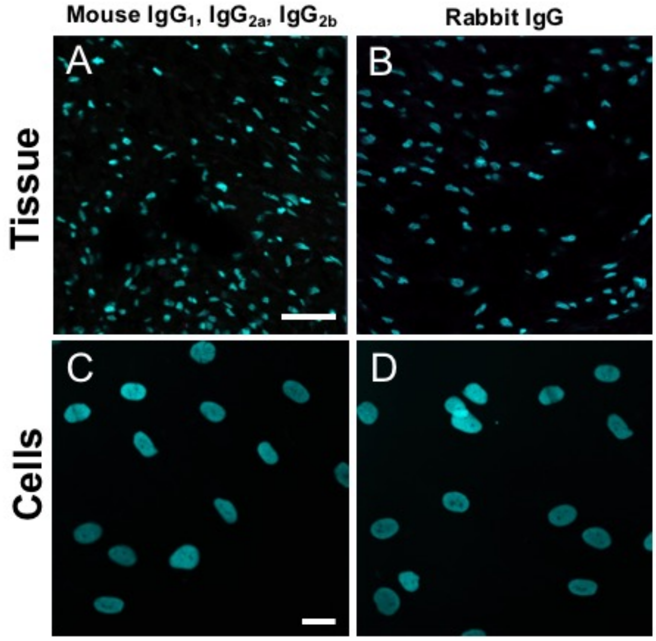

Figure S5. Isotype control staining of tissues and cells isolated from diseased human tendons. Representative confocal immunofluorescence images showing merged images of **(A, B)** diseased shoulder tendon tissue sections and **(C, D)** stromal cells isolated from diseased shoulder tendons stained with isotype control antibodies for mouse IgG_1, IgG_{2a}, IgG_{2b} and rabbit IgG fractions. Cyan represents POPO-1 nuclear counterstain. Scale bars, 20μm.

Supplement Table 1. 15-epi-LXA$_4$ and MaR1 up regulate SPM and reduce inflammation-initiating eicosanoids in healthy tendon stromal cells.

			Tendon stromal cells Lipid mediators levels (pg/incubation)			
**DHA bioactive metabolome**	**Q1**	**Q3**	**Healthy+ IL-1β**	**Healthy+ IL-1β + 0.1nM 15-epi-LXA$_4$**	**Healthy+ IL-1β + 10nM 15-epi-LXA$_4$**	**Healthy+ IL-1β + 10nM MaR1**
RvD1	375	141	9.8 ± 1.4	12.2 ± 2.2 *	18.8 ± 13.5	12.4 ± 3.0 *
RvD2	375	141	18.3 ± 5.4	36.7 ± 12.9	16.8 ± 8.6	20.7 ± 10.0
RvD3	375	147	9.3 ± 3.3	13.9 ± 1.3	10.8 ± 5.7	10.9 ± 6.5
RvD4	375	101	9.1 ± 2.5	16.4 ± 3.5	16.3 ± 7.6	11.3 ± 5.5
RvD5	359	199	60.0 ± 1.7	63.5 ± 1.0 *	106.7 ± 61.8	69.6 ± 9.1
RvD6	359	101	8.9 ± 1.5	13.9 ± 1.6 *	40.0 ± 38.5	17.8 ± 4.4
17R-RvD1	375	141	3.6 ± 0.1	4.5 ± 1.0	5.8 ± 3.0	2.7 ± 1.4
17R-RvD3	375	147	9.1 ± 1.9	12.3 ± 4.3	9.5 ± 1.4	10.5 ± 4.4
PD1	359	153	37.2 ± 6.4	40.4 ± 9.4	41.8 ± 7.5	37.3 ± 18.1
17R-PD1	359	153	1.3 ± 0.1	1.1 ± 0.1	2.6 ± 2.1	1.2 ± 0.2
10S,17S-diHDHA	359	153	157.7 ± 5.9	146.6 ± 19.4	185.7 ± 13.4 *	134.4 ± 33.5
MaR1	359	221	9.4 ± 2.8	10.3 ± 2.0 *	16.9 ± 4.5	525.5 ± 158.1 **
MaR2	359	191	2.1 ± 0.9	4.1 ± 2.9	3.6 ± 1.7	2.5 ± 2.2
7S,14S-diHDHA	359	221	35.6 ± 2.8	39.5 ± 3.2	42.0 ± 10.8	36.3 ± 13.8
4S,14S-diHDHA	359	101	3.4 ± 1.2	6.7 ± 1.5 *	4.8 ± 2.8	4.2 ± 2.3
14-oxo-MaR1	357	248	2.9 ± 1.1	0.0 ± 0.0	3.0 ± 1.7	19.5 ± 9.2 *
22-OH-MaR1	375	221	8.4 ± 2.5	19.1 ± 3.9	11.3 ± 6.5	22.7 ± 9.5 *
**n-3 DPA bioactive metabolome**						
RvD1$_{n-3\ DPA}$	377	143	1.6 ± 0.5	4.4 ± 2.4	10.7 ± 10.6	3.2 ± 2.1
RvD2$_{n-3\ DPA}$	377	261	1.4 ± 1.0	2.2 ± 0.5	1.9 ± 1.4	2.1 ± 1.0
RvD5$_{n-3\ DPA}$	361	263	17.2 ± 3.2	14.0 ± 0.6	46.7 ± 42.3	15.7 ± 2.6
PD1$_{n-3\ DPA}$	361	183	97.1 ± 32.7	113.6 ± 41.1	98.9 ± 67.3	90.6 ± 55.9
10S,17S-diHDPA	361	183	898.9 ± 241.5	1407.8 ± 98.3	1009.1 ± 480.8	967.5 ± 449.9
MaR1$_{n-3\ DPA}$	361	249	25.5 ± 6.8	34.2 ± 8.4	21.3 ± 9.1	26.2 ± 11.7
**EPA bioactive metabolome**						
RvE1	349	195	0.3 ± 0.1	0.4 ± 0.5	0.2 ± 0.3	0.6 ± 0.4
RvE2	333	199	38.7 ± 5.3	47.5 ± 11.2	48.2 ± 25.5	36.7 ± 18.1
RvE3	333	201	0.8 ± 0.5	1.3 ± 0.8	1.3 ± 0.8	1.2 ± 0.8
**AA bioactive metabolome**						
LXA$_4$	351	217	3.2 ± 1.5	333.2 ± 48.7 *	191.9 ± 80.8 *	268.8 ± 152.3 *
LXB$_4$	351	221	74.3 ± 25.6	278.0 ± 232.0	251.5 ± 130.2	119.5 ± 18.5
5S,15S-diHETE	335	235	1940.0 ± 187.3	2491.0 ± 209.4	2367.6 ± 404.2 *	2250.3 ± 282.3 *
15epi-LXA$_4$	351	217	186.2 ± 63.6	298.4 ± 48.7	655.9 ± 64.1 **	266.5 ± 140.8
15epi-LXB$_4$	351	221	80.0 ± 22.5	44.3 ± 11.8	79.4 ± 18.7	50.9 ± 3.2
15-oxo-LXA$_4$	349	233	10.7 ± 0.1	11.5 ± 3.7	30.6 ± 4.7 **	12.1 ± 2.6
LTB$_4$	335	195	0.0 ± 0.0	0.0 ± 0.0	0.0 ± 0.0	0.0 ± 0.0
5S,12S-diHETE	335	195	288.3 ± 74.0	436.1 ± 45.6 *	338.3 ± 143.8	301.0 ± 136.1
PGD$_2$	351	189	2465.9 ± 1459.0	322.4 ± 62.7 *	2086.3 ± 2506.7 *	2012.6 ± 2049.3 *
PGE$_2$	351	189	27091.1 ± 10813.8	27436.1 ± 13292.2	21140.3 ± 14581.1 *	21214.6 ± 15739.8 *
PGF$_{2\alpha}$	353	193	540.0 ± 81.6	468.1 ± 170.3	485.7 ± 97.3	420.8 ± 97.6
TxB$_2$	369	169	20.6 ± 3.6	15.4 ± 1.3	21.0 ± 6.7	15.9 ± 3.2

Tendon stromal cells (60,000 cells per well) were derived from healthy hamstring tendons. Cells were incubated for 24h (37°C) with 15-epi-LXA$_4$ (0.1 or 10 nM) or MaR1 (10nM) then with IL-1β (37°C; 24 h). Incubations were quenched using ice-cold methanol containing deuterium labelled internal standards and lipid mediators (LM) were identified and quantified using LM-profiling (see methods for details). Q1, M-H (parent ion) and Q3, diagnostic ion in the MS-MS (daughter ion). Results are expressed as pg/incubation. Mean ± SEM of n=7 per incubation. *P < 0.05, **P <0.01 vs Healthy+IL1β incubations. The detection limit was ~ 0.1 pg. -, Below levels found in media alone.

Supplement Table 2. Regulation of lipid mediator profiles by 15-epi-LXA_4 and MaR1 in diseased tendon stromal cells.

			Tendon stromal cells Lipid mediators levels (pg/incubation)			
**DHA bioactive metabolome**	**Q1**	**Q3**	**Diseased + IL-1β**	**Diseased + IL-1β + 0.1nM 15-epi-LXA_4**	**Diseased + IL-1β + 10nM 15-epi-LXA_4**	**Diseased + IL-1β + 10nM MaR1**
RvD1	375	141	5.6 ± 4.4	11.1 ± 2.8 *	6.2 ± 4.4	5.6 ± 4.7 *
RvD2	375	141	15.1 ± 11.4	30.1 ± 9.7	14.7 ± 10.4	17.4 ± 13.1
RvD3	375	147	5.7 ± 4.6	12.0 ± 2.6	5.3 ± 4.0	7.2 ± 5.4
RvD4	375	101	5.9 ± 3.8	13.1 ± 2.0	7.6 ± 3.9	6.9 ± 5.0
RvD5	359	199	46.4 ± 14.2	62.7 ± 12.2	48.2 ± 15.2	47.6 ± 12.3
RvD6	359	101	14.2 ± 4.8	15.4 ± 5.7	15.8 ± 5.4	13.9 ± 2.1
17R-RvD1	375	141	2.0 ± 1.1	2.5 ± 1.4	3.7 ± 1.5	3.2 ± 1.8
17R-RvD3	375	147	5.9 ± 3.0	13.2 ± 5.4	7.2 ± 2.8	6.8 ± 3.6
PD1	359	153	28.0 ± 22.4	48.5 ± 25.1	28.4 ± 23.9	22.9 ± 20.5
17R-PD1	359	153	0.6 ± 0.4	1.5 ± 0.1	0.8 ± 0.6	0.7 ± 0.5
10S,17S-diHDHA	359	153	117.1 ± 82.6	189.7 ± 48.2	129.4 ± 50.5	112.9 ± 54.1
MaR1	359	221	21.0 ± 14.7	15.6 ± 4.0	11.9 ± 4.3	346.0 ± 138.0
MaR2	359	191	8.2 ± 6.1	14.5 ± 3.9	9.7 ± 6.8	10.0 ± 7.5
7S,14S-diHDHA	359	221	26.4 ± 12.7	28.1 ± 11.1	23.6 ± 9.2	25.1 ± 9.4
4S,14S-diHDHA	359	101	3.6 ± 3.5	9.5 ± 3.6	3.9 ± 3.6	3.2 ± 2.6
14-oxo-MaR1	357	248	0.0 ± 0.0	0.0 ± 0.0	1.1 ± 1.1	46.8 ± 11.2
22-OH-MaR1	375	221	6.0 ± 2.8	10.4 ± 4.5	7.2 ± 3.6	4.8 ± 2.7
**n-3 DPA bioactive metabolome**						
$RvD1_{n\text{-}3\ DPA}$	377	143	4.5 ± 1.8	6.9 ± 2.3	6.2 ± 2.4	4.1 ± 0.8
$RvD2_{n\text{-}3\ DPA}$	377	261	0.9 ± 0.6	1.9 ± 0.5	1.1 ± 1.1	1.0 ± 0.6
$RvD5_{n\text{-}3\ DPA}$	361	263	15.8 ± 6.8	16.1 ± 3.2	15.0 ± 5.5	13.2 ± 2.2
$PD1_{n\text{-}3\ DPA}$	361	183	98.6 ± 108.0	157.6 ± 97.8	93.7 ± 91.5	76.9 ± 86.6
10S,17S-diHDPA	361	183	657.7 ± 573.4	1534.8 ± 483.6	683.3 ± 591.3	535.9 ± 420.9
$MaR1_{n\text{-}3\ DPA}$	361	249	12.7 ± 8.5	25.3 ± 13.0	13.1 ± 9.3	12.1 ± 8.8
**EPA bioactive metabolome**						
RvE1	349	195	0.1 ± 0.2	0.1 ± 0.1	0.1 ± 0.1	0.1 ± 0.1
RvE2	333	199	55.7 ± 13.6	74.0 ± 5.4	59.7 ± 18.4	45.3 ± 8.9
RvE3	333	201	0.7 ± 0.6	2.6 ± 0.1	2.1 ± 1.7	0.9 ± 0.6
**AA bioactive metabolome**						
LXA_4	351	217	3.4 ± 3.5	37.2 ± 19.3	142.5 ± 106.2	241.0 ± 200.0
LXB_4	351	221	58.0 ± 57.3	109.0 ± 40.4	117.0 ± 96.8	90.0 ± 59.1
5S,15S-diHETE	335	235	1752.2 ± 1034.1	3004.4 ± 866.9	1815.1 ± 916.1	1487.7 ± 821.3
15epi-LXA_4	351	217	287.6 ± 165.1	427.1 ± 92.1	473.4 ± 124.1	256.0 ± 200.4
15epi-LXB_4	351	221	55.5 ± 22.6	53.0 ± 4.7	88.2 ± 42.2	73.9 ± 10.4
15-oxo-LXA_4	349	233	2.5 ± 2.7	9.3 ± 0.2	55.4 ± 11.2	5.2 ± 4.9
LTB_4	335	195	0.0 ± 0.0	0.0 ± 0.0	0.0 ± 0.0	0.0 ± 0.0
5S,12S-diHETE	335	195	232.1 ± 201.8	512.3 ± 182.1	242.4 ± 197.6	214.3 ± 162.6
PGD_2	351	189	1161.0 ± 628.4	231.5 ± 92.9	176.9 ± 72.9	145.0 ± 78.1
PGE_2	351	189	23342.7 ± 18984.9	39971.2 ± 10694.7	22697.5 ± 17442.7	22088.1 ± 17520.2
$PGF_{2\alpha}$	353	193	300.7 ± 206.0	416.7 ± 234.1	269.0 ± 185.5	293.2 ± 224.6
TxB_2	369	169	10.1 ± 4.1	14.6 ± 1.7	8.5 ± 2.4	9.1 ± 2.3

Tendon stromal cells (60,000 cells per well) were derived from diseased supraspinatus tendons. Cells were incubated for 24h (37°C) with 15-epi-LXA_4 (0.1 or 10 nM) or MaR1 (10nM) then with IL-1β (37°C; 24 h). Incubations were quenched using ice-cold methanol containing deuterium labelled internal standards and lipid mediators (LM) were identified and quantified using LM-metabololipidomics (see methods for details). Q1, M-H (parent ion) and Q3, diagnostic ion in the MS-MS (daughter ion). Results are expressed as pg/incubation. Mean ± SEM of n=6 per incubation. *P < 0.05, **P <0.01 vs Disease+IL1β incubations. The detection limit was ~ 0.1 pg. -, Below levels found in media alone.
